# Single-cell mRNA sequencing identifies subclonal heterogeneity in anti-cancer drug responses of lung adenocarcinoma cells

**DOI:** 10.1186/s13059-015-0692-3

**Published:** 2015-06-19

**Authors:** Kyu-Tae Kim, Hye Won Lee, Hae-Ock Lee, Sang Cheol Kim, Yun Jee Seo, Woosung Chung, Hye Hyeon Eum, Do-Hyun Nam, Junhyong Kim, Kyeung Min Joo, Woong-Yang Park

**Affiliations:** Samsung Genome Institute, Samsung Medical Center, Seoul, South Korea; Institute for Refractory Cancer Research, Samsung Medical Center, Seoul, South Korea; Department of Urology, Samsung Medical Center, Sungkyunkwan University, Seoul, South Korea; Department of Neurosurgery, Samsung Medical Center, Sungkyunkwan University, Seoul, South Korea; Department of Anatomy and Cell Biology, Sungkyunkwan University School of Medicine, Seoul, South Korea; Department of Molecular Cell Biology, Sungkyunkwan University School of Medicine, Seoul, South Korea; Department of Health Sciences and Technology, SAIHST, Sungkyunkwan University, Seoul, South Korea; Department of Biomedical Sciences, College of Medicine, Seoul National University, Seoul, South Korea; Department of Biology, University of Pennsylvania, Philadelphia, PA 19104 USA; Penn Program in Single Cell Biology, University of Pennsylvania, Philadelphia, PA 19104 USA

## Abstract

**Background:**

Intra-tumoral genetic and functional heterogeneity correlates with cancer clinical prognoses. However, the mechanisms by which intra-tumoral heterogeneity impacts therapeutic outcome remain poorly understood. RNA sequencing (RNA-seq) of single tumor cells can provide comprehensive information about gene expression and single-nucleotide variations in individual tumor cells, which may allow for the translation of heterogeneous tumor cell functional responses into customized anti-cancer treatments.

**Results:**

We isolated 34 patient-derived xenograft (PDX) tumor cells from a lung adenocarcinoma patient tumor xenograft. Individual tumor cells were subjected to single cell RNA-seq for gene expression profiling and expressed mutation profiling. Fifty tumor-specific single-nucleotide variations, including *KRAS*^*G12D*^, were observed to be heterogeneous in individual PDX cells. Semi-supervised clustering, based on *KRAS*^*G12D*^ mutant expression and a risk score representing expression of 69 lung adenocarcinoma-prognostic genes, classified PDX cells into four groups. PDX cells that survived *in vitro* anti-cancer drug treatment displayed transcriptome signatures consistent with the group characterized by *KRAS*^*G12D*^ and low risk score.

**Conclusions:**

Single-cell RNA-seq on viable PDX cells identified a candidate tumor cell subgroup associated with anti-cancer drug resistance. Thus, single-cell RNA-seq is a powerful approach for identifying unique tumor cell-specific gene expression profiles which could facilitate the development of optimized clinical anti-cancer strategies.

**Electronic supplementary material:**

The online version of this article (doi:10.1186/s13059-015-0692-3) contains supplementary material, which is available to authorized users.

## Background

Identification of somatic driver mutations in cancer has led to the development of targeted therapeutics that have improved the clinical outcomes of cancer patients [1–3]. Lung adenocarcinoma (LUAD), the most common histological subtype of non-small cell lung cancer [4], is denoted by genetic alterations in the receptor tyrosine kinase (RTK)-RAS-mitogen-activated protein kinase (MAPK) pathway [2]. Companion diagnostics for hotspot mutations of EGFR, KRAS, BRAF, and ALK, which are clinically associated with specific targeted cancer therapies, are currently available for LUADs [5]. While the detection rate of currently identified actionable mutations in LUAD is over 60 % [2], efforts to catalogue all the clinically relevant genetic variations are still ongoing [6–9]. Moreover, drug resistance and disease recurrence after anti-cancer treatments require more comprehensive genomic analysis of individual LUADs [10, 11].

Although the individual cells in a tumor mass originate from a common ancestor and share early tumor-initiating genetic alterations, tumor cells frequently diverge and show heterogeneity in growth [12–14], drug resistance [15, 16], and metastatic potential [13, 14]. Intra-tumoral heterogeneity results from mutation and clonal selection dynamics during tumor growth [13, 14, 16], where individual tumor cells accumulate cell-specific genetic changes [12]. This genetic heterogeneity is significantly associated with tumor progression and the treatment outcomes of cancers [17, 18]. Therefore, monitoring intra-tumoral heterogeneity at the single-cell level would broaden our understanding of tumor recurrence mechanisms after anti-cancer treatments [19] and guide us in developing more sophisticated strategies to overcome drug resistance.

Single-cell genome profiling technology provides the highest-resolution analysis of intra-tumoral genetic heterogeneity [20–22]. Based on heterogeneity, we can identify individual cells with specific genetic alterations or genomic expression profiles that could be responsible for treatment resistance. Therefore, correlating the genotype–phenotype relationship in genetically distinct single cells can provide important new information for selecting the most appropriate clinical intervention for targeting heterogeneous LUADs [23]. For this purpose, patient-derived xenograft (PDX) cells provide a genetically and phenotypically accessible model for single cancer cell analyses of the heterogeneous histopathological, genetic, molecular, and functional characteristics of parental tumors [24, 25]. Moreover, drug-resistant tumor cells can be selected and analyzed *in vitro* using PDX cells.

We performed transcriptome profiling on single PDX cells from a LUAD patient to elucidate the molecular mechanisms and underlying genomic characteristics of tumor cell resistance to anti-cancer drug treatments. Single-cell transcriptome analysis uncovered heterogeneous behaviors of individual tumor cells and provided new insights into drug resistance signatures that were masked in bulk tumor analyses.

## Results

### Intra-tumoral genetic heterogeneity of LUAD PDX cells

**Fig. 1:**
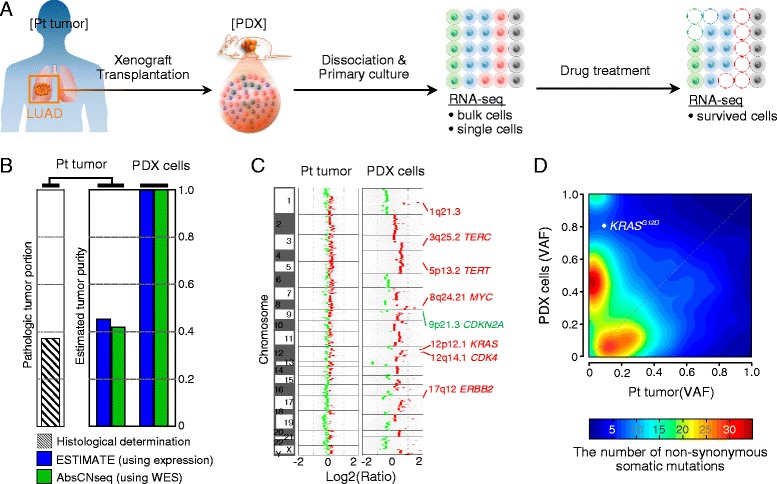
Enrichment of cancer cells in the PDX. **a** Schematic representation of experiments. A portion of a LUAD patient tumor (*Pt tumor*) was propagated by xenograft transplantation in humanized immunocompromised female NOG mice. PDX cells (*PDX*) were dissociated and cultured from xenograft tumors, and subjected to drug screening. **b** Estimated cancer cell fraction in Pt tumor and PDX cells. The fraction was quantified by histopathological examination (*striped bar*), or estimated based on computational analysis using expression profiles (*blue*) or WES data (*green*). **c** Estimated degree of normalized copy number changes in log2 ratio to matched peripheral blood for deletion (*green*) or amplification (*red*) are indicated. Representative sites of copy number changes in LUAD are labeled on the right side. **d** Distribution of variant allele frequencies (*VAF*) of the non-synonymous somatic mutations that overlap between Pt tumor and PDX cells. Color-scaled density map indicates the number of mutations

Surgically removed LUAD tissue was propagated through xenograft engraftments in mice (Fig. 1a). Viable cancer cells were dissociated from the PDX tissue and primarily cultured *in vitro* (Figure S1a in Additional file 1). Cultured PDX cells were genomically analyzed by RNA sequencing (RNA-seq) and whole-exome sequencing (WES). Although the tumor portion in the surgical sample represented approximately 40 % of the excised tissue volume (Figure S1b in Additional file 1), multiple validated genomic analyses utilizing WES [26, 27] and expression profiles [28] indicated that human cancer cells were highly enriched (~100 %) in the PDX cells (Fig. 1b). Overall, copy number alterations and variant allele frequencies were increased in the PDX tumor, compared with the surgical specimen (Fig. 1c, d). Some mutations present in the patient tumor were lost in the PDX, suggesting that our PDX model went through a selective engraftment process [29]. The histologic characteristics of the patient tumor were well preserved in the PDX (Figure S1c in Additional file 1). The full profiles of somatic mutations in the patient tumor and PDX cells are listed in Additional file 2.

Tumor cell-enriched PDX cells (LC-PT-45) [30] were further analyzed by single-cell RNA-seq using the Fluidigm C1™ autoprep system with SMART-seq [31]. cDNAs from 34 individual PDX cells were successfully amplified. Using 100-bp paired-end sequencing, we obtained an average of 8.12 ± 2.34 million mapped reads from the captured cells (Additional file 3). Overall, 85.63 % of reads mapped to the human reference genome, which was a lower percentage than is typical for unamplified conventional RNA-seq, but comparable to other single cell RNA-seq data [31, 32]. We also sequenced 50 single H358 human lung cancer cells as cell line controls and obtained an 85.39 % mapping rate (Additional file 3). Noticeably skewed coverage at the 3’ end of transcripts, which was inversely proportional to the expression level, was observed in the single-cell RNA-seq data (Additional file 4). The use of smaller initial RNA templates for amplification is known to increase this bias [31].

**Fig. 2:**
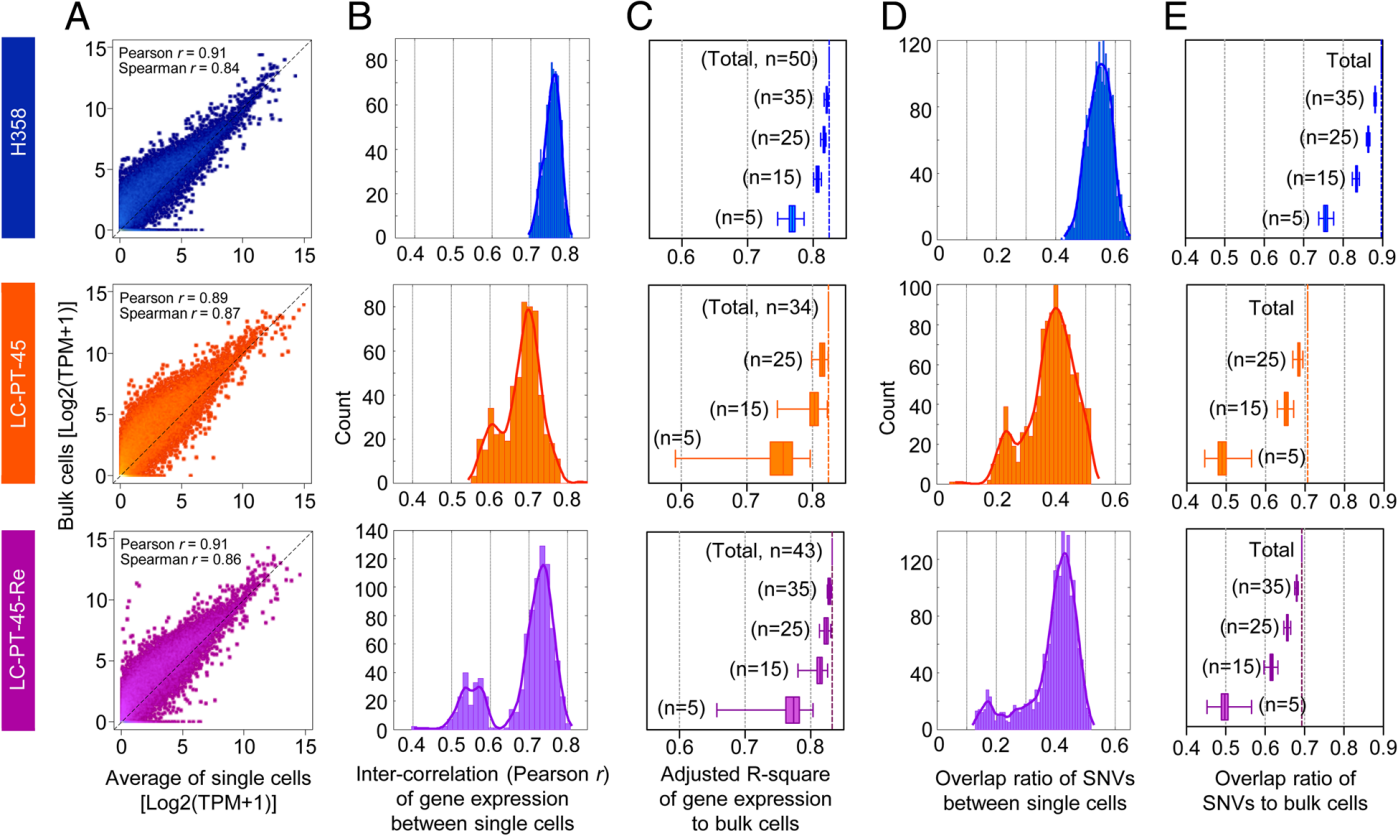
Intra-tumoral heterogeneity of PDX cells. **a** Scatter plots of the average gene expression of single cells (H358, n = 50; LC-PT-45, n = 34; LC-PT-45-Re, n = 43) compared with those of the corresponding bulk cells (~1 × 10^5^ cells). *Black dotted lines* are *x = y* lines with correlation coefficients (Pearson *r* and Spearman *r*) for linear fit. *TPM* transcripts per million. **b** Inter-correlation (Pearson *r*) between gene expression in single cells. Density plots were constructed with a kernel function fitting over the histograms. **c** Explanatory power (adjusted R-square) of gene expression in various numbers of single cells relative to the bulk cells was determined by multiple regression analysis with randomly selected cell numbers with permutation (×1000). **d** Overlap ratio of expressed single-nucleotide variations (*SNV*) among single cells. Density plots were constructed with a kernel function fitting over the histograms. **e** Overlap ratio of expressed SNVs of various numbers of single cells relative to that of the bulk cells was calculated with a randomly selected given number of cells with permutation (×1000). For boxplots in (**c**) and (**e**), box = interquartile range between the first and the third quartiles, error bars = 10th–90th percentiles

Despite the sequencing bias in amplified RNAs, average gene expression in single cells correlated well with expression in bulk cells, for both H358 and PDX cells (Fig. 2a). The inter-correlation of total gene expression among the 34 individual PDX cells showed wider distribution compared with that in the 50 H358 cells (Fig. 2b), indicating moderately higher transcriptome heterogeneity. The level of transcriptome heterogeneity was also evaluated by multiple regression analysis of different sized pools (n = 5, 15, 25, 34/35, 50; randomly selected by permutation × 1000) of single cell transcriptomes to the bulk sample (Fig. 2c). The modeling demonstrated that five H358 or PDX individual cells represented >70 % of the gene expression of the whole population. When averaging increased numbers of cells, the single cell data approximated the bulk up to 85 %, suggesting that the single cell data are consistent with the bulk data (Fig. 2c). We repeated the single cell isolation and RNA-seq using 43 additional PDX cells and obtained comparable results that were highly correlated with the first data set (Fig. 2; Figure S3a–f in Additional file 5, LC-PT-45 and LC-PT-45-Re). Comparisons of gene expression data for the 43 target genes (see Additional file 6 for the gene list and Figure S3g in Additional file 5 for expression levels) between technical replicate RNA-seq sets (Figure S3h left in Additional file 5) or between RNA-seq and quantitative PCR (qPCR) analysis (Figure S3h right in Additional file 5) also demonstrated statistically significant correlation, comparable to that reported in a previous publication [33].

### Single-cell heterogeneity of expressed single-nucleotide variants

To estimate tumor heterogeneity at the genetic mutation level, we identified expressed single-nucleotide variants (SNVs) using the single-cell RNA-seq and bulk WES data (Figure S4a in Additional file 7). After removal of potential false positive SNVs specifically found in RNA-seq using the SNPiR package [34], higher overlap ratios to bulk WES data were observed (Figure S4b middle panels in Additional file 7). Selection of SNVs found in both single cell RNA-seq and bulk WES data significantly increased the overlap ratios to dbSNP137 (Figure S4b right panels in Additional file 7). These filtered SNVs of individual PDX cells showed relatively heterogeneous expression compared with those of H358 cells in terms of the lower overlap ratios between single cells (Fig. 2d). The union of SNVs from five PDX cells (randomly selected by permutation × 1000) reflected 49 % of the expressed SNVs in the whole population, whereas those of five H358 cells represented 75 % (Fig. 2e). With increased numbers of single cells, the coverage increased up to 70 and 90 % for PDX cells (34 LC-PT-45 or 43 LC-PT-45-Re) and H358 cells, respectively.

**Fig. 3:**
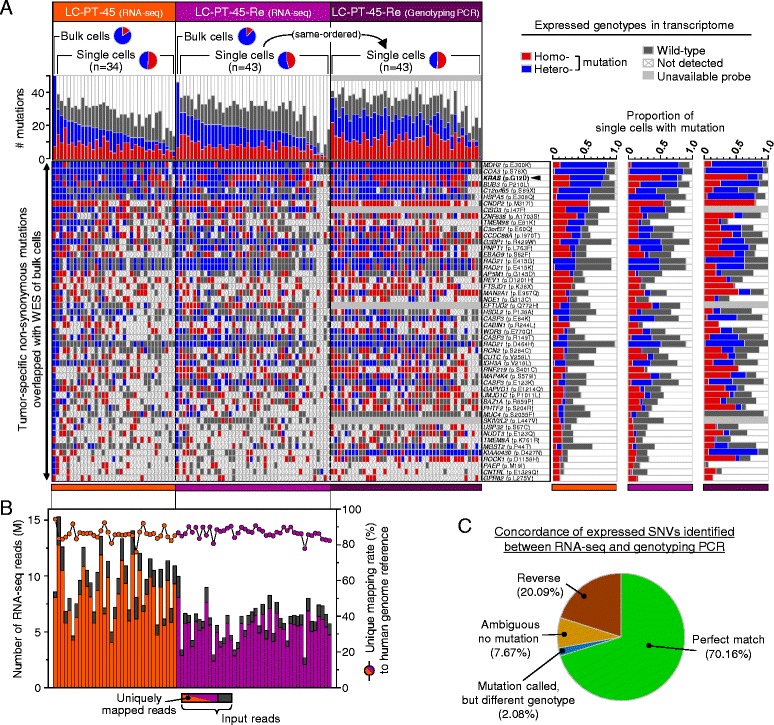
Heterogeneous expression patterns of SNVs in PDX cells. **a** Expressed, tumor-specific, non-synonymous somatic mutations found in more than three single cells of LC-PT-45. The replicate batch (LC-PT-45-Re) of single-cell RNA-seq and that of genotyping PCR are shown together. The bar graphs at top left show mutation events per sample; the heat maps at bottom left show mutation profiles across samples; the bar graphs at the right show normalized mutation fraction over total single cells (LC-PT-45, n = 34; LC-PT-45-Re, n = 43). **b** Mapping information from RNA-seq for reads mapped to the human reference genome (hg19). The bar graph (left y-axis) shows the number of RNA-seq reads and the scatter plots (right y-axis) with a connecting line show the unique mapping rate (uniquely mapped reads/input reads) in the same order as in (**a**). **c** Summary of results for the matched samples and the validated targets between RNA-seq and genotyping PCR shown in (**a**). See Additional file 8 for the details

After exclusion of germline variants by selecting only somatic SNVs from bulk WES data, expression of 50 tumor-specific non-synonymous SNVs was analyzed in individual PDX cells (Figure S4a in Additional file 7). The 50 tumor-specific SNVs showed heterogeneous expression patterns in the individual PDX cells (Fig. 3a, LC-PT-45) with numerous allele dropouts. For comparison, we plotted expression of lung cancer mutations in the H358 cell line listed in COSMIC [35] (Figure S5a in Additional file 8), which also showed variable expression patterns with more uniform coverage (Figure S5b in Additional file 8). For the PDX cells, we detected comparable mutation patterns and frequencies in the original and replicate PDX analyses (Fig. 3a; Figure S3c, f in Additional file 5, LC-PT-45 vs. LC-PT-45-Re RNA-seq). The number of reads mapped to the human genome reference were determined for individual cells to assure sequencing quality (Fig. 3b). We also performed genotyping PCR on the LC-PT-45-Re samples in parallel, which showed >70 % concordance with the RNA-seq results [Fig. 3a, LC-PT-45-Re (RNA-seq) vs. LC-PT-45-Re (genotyping PCR), and Fig. 3c; Additional file 9]. Together these data support reproducible cellular variance in SNV expression. Nevertheless, no calls and discrepant mutation calls between RNA-seq and genotyping PCR demonstrate limitations of single cell RNA-seq, which might have originated from allelic dropouts.

Among the genes with SNVs detected in PDX cells, *KRAS* [1, 2], *GAPVD1* [36], and *JMJD1C* [37] are functionally related to the RTK-RAS-MAPK signaling pathway. The hotspot *KRAS*^*G12D*^ mutation was detected in 27 out of 34 single PDX cells (79.4 %), or 33 out of 43 PDX replicates (76.7 %). To determine whether the variable mutant allele expression was due to genetic heterogeneity, we assessed the genotypes of 12 somatic mutations at the single-cell DNA level with droplet digital PCR (ddPCR; Figure S7a in Additional file 10). When mutation rates were computed as variant allele frequencies in bulk cells or as mutant single cell fractions at both the DNA and RNA levels, they showed overall correlation (Figure S7b in Additional file 10). With respect to the *KRAS* mutation, all PDX cells (21 of 21) harbored the mutant allele in the single-cell DNA analysis. Of note, copy number gains (Fig. 1c) and mutant/wild-type ratios in *KRAS* (Figure S7c in Additional file 10) suggest that variable copy numbers of the mutant KRAS influenced the differential allele expression. These data suggest that genetic heterogeneity contributes to variable mutant allele expression. In addition, allele-biased expression may also contribute to mutant allele expression heterogeneity. Given the importance of oncogenic *KRAS* mutations, we defined two subpopulations in the PDX based on the expressed genotype: one with dominant *KRAS*^*G12D*^ expression, and another without *KRAS*^*G12D*^ expression (*KRAS*^*wild type (WT)*^ expression).

### Identification of PDX cell subgroups

**Fig. 4:**
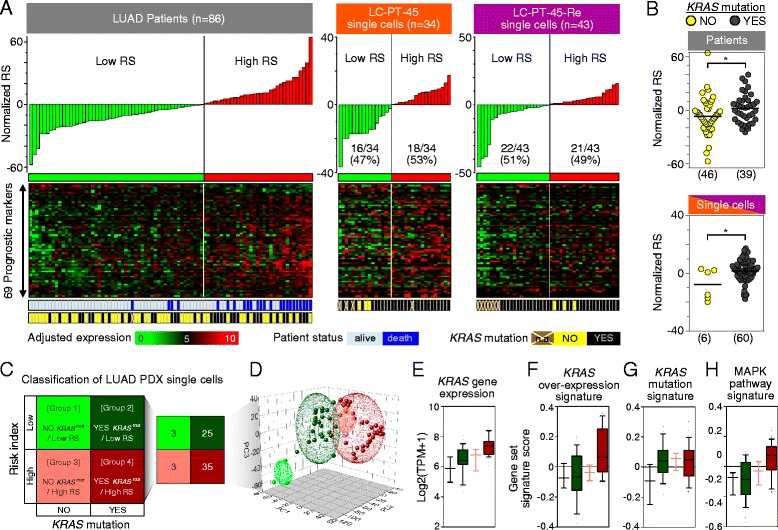
Identification of PDX cell subclones using single-cell RNA-seq data. **a** *Top*: normalized RS. *Middle*: heatmaps of expression of 69 prognostic markers. *Bottom*: *KRAS* mutation status of each patient (training set, n = 86) or single cell (LC-PT-45, n = 34; LC-PT-45-Re, n = 43). **b** Scatter plots demonstrating the effect of the *KRAS* mutation on the RSs of LUAD patients and PDX single cells. *Horizontal lines* represent the mean. *P < 0.05; **P < 0.01. **c** Semi-supervised clustering of single cells into four groups with estimated RS and *KRAS* mutant status. **d** Principal component analysis of the genes discriminating the subgroups. Ellipsoids were generated with standard deviations around each group. **e**–**h** Comparative features among the classified single cell subgroups. **e** *KRAS* gene expression (Log2 ratio of transcripts per million + 1). Gene set signature scores (computed by gene set variation analysis) corresponding to the *KRAS* over-expression signature [39] (**f**), *KRAS* mutation signature [40] (**g**), and MAPK pathway signature (gene sets from BioCarta) (**h**). For the boxplots in (**e**–**h**), boxes = the interquartile range between the first and third quartiles, and error bars = 10th–90th percentiles

To further identify subclones with possible phenotypic implications in the PDX cells, we utilized the expression profiles of 69 genes related to the clinical prognosis of LUAD patients (Additional file 11) [6] as multivariate markers to compute a risk score (RS) (Fig. 4a). A previous study [6] defined a high-RS population as those with the top 40 % of RSs (normalized RS > 0). The prognostic significance of the RS was validated in two independent public datasets from The Cancer Genome Atlas and from Korean LUAD patients (Additional file 12). Moreover, a higher RS was significantly associated with the *KRAS* mutation in the LUAD patient population [6] (Fig. 4b), which is consistent with a previously observed correlation of the *KRAS* mutation with worse clinical outcomes [5, 38].

Interestingly, individual PDX cells were calculated to have a wide RS distribution (Fig. 4a). Eighteen out of the 34 PDX cells or 21 out of 43 of the replicate samples were determined to be high-RS. We combined the replicate PDX RNA-seq data for further analysis and found that PDX cells with *KRAS*^*G12D*^ expression tend to have a higher RS (Fig. 4b). The finding is consistent with those of LUAD patients in clinical studies [6]. Altogether, semi-supervised clustering based on the expression of the *KRAS* mutation and RS classified the PDX cells into four groups: group 1, no *KRAS*^*G12D*^ (*KRAS*^*WT*^)/low RS (n = 3); group 2, *KRAS*^*G12D*^/low RS (n = 25); group 3, no *KRAS*^*G12D*^ (*KRAS*^*WT*^)/high RS (n = 3); and group 4, *KRAS*^*G12D*^/high RS (n = 35) (Fig. 4c).

These four groups displayed characteristic gene expression profiles that likely reflect the different phenotypes among individual PDX cells (Figure S9a in Additional file 13). In particular, group 4 had enhanced gene expression signatures associated with KRAS overexpression and activation of the RAS-MAPK signaling pathway [39, 40] (Fig. 4f, h), which correlated well with *KRAS* mutational status. Group 4 PDX cells also showed significantly higher cell cycle gene mRNA expression (Figure S9c in Additional file 13) [41]. In contrast, despite having the *KRAS* mutation signature (Fig. 4g), group 2 cells had lower KRAS expression levels and KRAS overexpression signatures (Fig. 4e, f), lower RAS-MAPK signaling pathway activation status (Fig. 4h), and reduced expression of cell cycle-related genes (Figure S9c in Additional file 13).

The distinct gene expression signatures among the four groups were visualized by a principal component analysis (PCA) plot using genes exclusively expressed by each group, with a criterion of at least a twofold change in transcripts per million (TPM) ratio with statistical significance (*t*-test *P* < 0.05; Fig. 4d). Although group 2 cells showed a lower RAS-MAPK signaling pathway activation status, they had significantly upregulated expression of ion channel transport pathway-related genes (Figure S9b in Additional file 13), which has been implicated in the drug resistance mechanism [10].

### Phenotypic interpretation of PDX cell subgroups

**Fig. 5:**
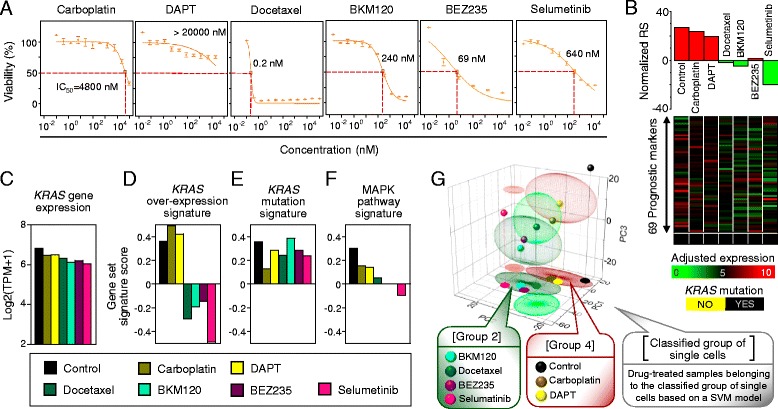
Interpretation of drug responses using single-cell signatures. **a** Dose response curves for the four selected anti-cancer compounds (cytotoxic compounds carboplatin and docetaxel; molecular targeting compounds DAPT, BKM120, BEZ235, and selumetinib). **b** Normalized RSs (*top*) and adjusted-expression of the 69 prognostic markers (*middle*) with *KRAS* mutant expression (*bottom*) for the control and drug-treated PDX cells. **c**–**f** Comparative features among the control and drug-treated PDX cells. **c** *KRAS* gene expression (Log2 ratio of TPM + 1). Gene set signature scores (computed by gene set variation analysis) corresponding to the *KRAS* over-expression signature [39] (**d**), *KRAS* mutation signature [40] (**e**), and MAPK pathway signature (gene sets from BioCarta) (**f**). **g** Results from the principal component (PC) analysis on single cells along with the control and drug-treated PDX cells. Ellipsoids correspond to the single cell subgroups (group 1, *light green*; group 2, *dark green*; group 3, *light red*; group 4, *dark red*), with the control and drug-treated PDX cells projected on the *PC1*-*PC2* plane. Using single cell subgroups as a training set, classification support vector machine type 1 (C-SVM classification) was applied to a test set of the control and drug-treated PDX cells

The results above indicated that, in the PDX cell population, there is a specific subgroup (group 4) that is predicted to be more aggressive than the other groups. This subset is characterized by a high RS, *KRAS* mutation, RAS-MAPK signaling pathway activation, and upregulation of cell cycle-related genes. To determine whether individual cells associate with tumor phenotypic aggressiveness, such as drug resistance, we screened the *in vitro* sensitivity of the PDX cells against a panel of 25 anti-cancer agents used in non-small cell lung cancer treatment (Additional file 14). The PDX cells were highly sensitive to a variety of drug treatments, including docetaxel, and molecular pathway targeting agents. Among the identified agents, we focused on the MEK1/2 inhibitor selumetinib, and the phosphatidylinositide 3-kinase (PI3K) inhibitors BKM120 and BEZ235 (PI3K/mTOR), because of their potential clinical benefits [42, 43]. Other cytotoxic drugs (e.g., carboplatin, and the Notch inhibitor DAPT) did not show any effects (Fig. 5a). Although docetaxel, BKM120, BEZ235, and selumetinib showed tumoricidal effects, some PDX cells survived the three days of treatment with these drugs when utilized at their reported IC_50_.

When evaluated as a bulk population, PDX cells manifested group 4-like characteristics with high RS and *KRAS*^*G12D*^. Ineffective treatments with carboplatin or DAPT did not alter these properties of the group (Fig. 5b–g). However, those PDX cells that survived the docetaxel, BKM120, BEZ235, or selumetinib treatments showed group 2-like gene expression signatures: low RS (Fig. 5b), slight decrease in total KRAS expression levels (Fig. 5c), down-regulation of gene expression signatures associated with KRAS overexpression (Fig. 5d), preservation of the mutant *KRAS*^*G12D*^ expression signature (Fig. 5e), and down-regulation of RAS-MAPK signaling pathway activation (Fig. 5f). Moreover, upregulation of ion channel transport genes (Figure S9b in Additional file 13) and downregulation of cell cycle-related genes (Figure S9c in Additional file 13) were observed in these treatment groups. The overall gene expression signature represented by PCA confirmed the group 2 cell-like properties of the drug-resistant PDX cells, in a support vector machine (SVM) model (Fig. 5g). Altogether, these results suggest that the drug-resistant population was cell-cycle quiescent and with possibly higher transporter activity for the anti-cancer drugs.

We further determined whether the group 2-like population conveyed the low risk gene expression signature after anti-cancer drug treatment with selumetinib (Figure S11a in Additional file 15). Interestingly, the low RS of surviving PDX cells was gradually reverted to a high RS after drug removal (Figure S11b in Additional file 15). The KRAS over-expression signature (Fig. S11d in Additional file 15) and MAPK pathway activation (Figure S11f in Additional file 15) recovered as well. By contrast, the level of total KRAS expression (Figure S11c in Additional file 15) and mutational status (Figure S11e in Additional file 15) were not altered by drug removal. The possible mechanisms of the dynamic nature of these gene expression signatures, such as epigenetic regulation or recovery of heterogeneity by clonal proliferation, need to be further elucidated.

### Validation of analytical procedures in an independent lung cancer PDX case

To validate our strategy of using single cell RNA-seq data for subgroup identification, we used an independent set of PDX cells derived from a lung cancer-brain metastasis (LC-MBT-15) [30]. The LC-MBT-15 PDX harbors an insertional mutation in *EGFR* exon 20, a well-known driver mutation in LUAD conferring resistance to reversible EGFR inhibitors [44, 45]. Single cells from LC-MBT-15 had less heterogeneous transcriptome and SNV expression compared with the *KRAS* mutant PDX cells (Figure S12a–e in Additional file 16), which might have been caused by extensive clonal selection during serial anti-cancer treatments before PDX establishment (see the patient description in “Materials and methods”). Nonetheless, the LC-MBT-15 single cells were still clustered into two subgroups by RS, similar to the original PDX case (Figure S12f, i in Additional file 16). In contrast to the *KRAS*^*G12D*^ mutation, the *EGFR* mutation was modestly detected and showed no preferential expression in the high RS group (Figure S12g, h in Additional file 16).

Drug screening on LC-MBT-15 cells was performed using 28 lung cancer drugs (Additional file 17). LC-MBT-15 cells were highly sensitive to the irreversible EGFR/HER2 inhibitor afatinib and the c-Met inhibitor tivantinib but were resistant to the reversible EGFR inhibitor erlotinib. When gene expression profiles for the drug-resistant populations were analyzed 3 days later, PCA of the single cells and application of a SVM model for drug-treated populations revealed that the drug-resistant populations shared the gene expression signature of the low RS group (Figure S12j, l in Additional file 16). Interestingly, upregulation of ion channel transport genes was also noted in the drug-resistant populations (Figure S12k in Additional file 16), similar to the low risk group single cells. These results are consistent with the original LC-PT-45 PDX case, and further support the observation that (1) single cell profiles of a population reveal cells with drug-resistant signatures and (2) the drug-resistant population may come from a subset with higher transporter activity and low cell proliferation activity.

## Discussion

Single-cell genome analysis enables measurement of the extent of intra-tumoral heterogeneity, which may provide clues for solving problems such as cancer recurrence, metastasis, and drug resistance [46]. Single-cell RNA-seq can provide integrative information on both gene expression and somatic SNVs, which makes it a comprehensive tool to connect a cell’s genotype with its expression profile and phenotype. We used tumor cell-enriched LUAD PDX cells to define genomic signatures of individual tumor cells, and then verified the applicability of translating this information into biological cancer cell phenotypes such as drug responses.

When interpreting single cell RNA-seq data, the data quality needs to be considered, because of the high magnitude of amplification in the sequencing process. Sequence errors can be incorporated during the reverse transcription, cDNA amplification, and library construction processes, causing false positive mutation calls. RNA editing and monoallelic expression can also cause discrepancies between SNV calls from RNA and DNA sequencing. In this study, we focused on the RNA-seq SNVs that were simultaneously detected by WES and identified in more than three single cells. This approach would minimize the probability of false positive SNV calls. On the other hand, false negative SNV calls could result from missing reads at the mutant position in both DNA and RNA sequencing, which might be misinterpreted as biological heterogeneity [47]. Various approaches such as Nuc-seq, which increases the starting material by using G2/M phase cells, are reported to increase the genome coverage up to 91 % for DNA sequencing [48]. For the RNA-seq-based genotype analysis, mutations in rare transcripts are most prone to the dropout events, suggesting that RNA-seq is suitable for genotyping highly expressed oncogenic driver mutations.

Despite limitations in the accuracy of single-cell RNA-seq, in this study we observed good correlations between the merged single-cell data and the bulk cell data at both the gene expression and expressed SNV levels. Once the number of single cells exceeded 30, the averaged expression levels and consensus SNVs largely recapitulated the data from bulk populations. Significant correlations were also detected between replicate RNA-seq analyses and with the PCR-based genotyping method. While these concordant results and overall high expression level of *KRAS* support the validity of the *KRAS* mutation calls in RNA, 12–16 % of cells had insufficient RNA read counts at the mutant position, resulting in ambiguous calls. Downsizing sequencing data further increased the number of ambiguous calls (data not shown), indicating that a sufficient number of reads is critical in the RNA-based mutation analysis. This is in contrast to the gene expression analysis, which required only 0.5 million reads for the transcriptome estimation [33].

We isolated single PDX cells from a *KRAS*-driven tumor, which represents 25–33 % of LUADs [2, 3]. Comparison of the patient tumor and PDX cells revealed a significant enrichment of *KRAS* mutant tumor areas in the PDX, indicating that this PDX is a good model in which to study *KRAS*-driven tumors. However, we observed loss of some mutations as well as increased variant allele frequencies of many shared mutations and de novo mutations, possibly resulting from the expansion of subclones [29]. These subclones might be undergoing proliferation due to clonal selection and adaptation in the PDX, leading to a transient increase in genetic variation of the sample. In the longer term, the selection would have diminished the level of tumor heterogeneity originally present in the patient tumor. Therefore, use of freshly isolated tumor cells is warranted for the accurate estimation of tumor heterogeneity in future studies.

Because activating *KRAS* mutations are associated with poor LUAD prognosis and due to the current lack of reliable targeting agents [5, 38], it is a clinical challenge to find efficient treatment strategies for *KRAS*-driven cancers. According to the *KRAS* mutation status, the PDX cells analyzed as a bulk population showed clinically unfavorable genomic characteristics when the RS was calculated from the signature of 69 prognostic genes [6]. However, single-cell RNA-seq of PDX cells revealed intra-tumoral heterogeneity in terms of the *KRAS* mutant and RS gene expression characteristics. Having individual tumor cells that display intra-tumorally heterogeneous molecular signatures that are prognostic in LUAD patients is an interesting attribute. Similar findings were reported in other single-cell or multi-regional studies in glioblastoma [32], in which single cells from the same tumor were classified into multiple subtypes. Moreover, glioblastoma patients with mixed subtype cells manifested worse prognoses [32], suggesting the prognostic value of defining intra-tumoral heterogeneity.

The intra-tumoral heterogeneity might be driven by DNA mutations as well as by epigenetic and regulatory mechanisms. In this study we identified individual cells with variable mutant *KRAS* gene expression and RSs. Both genetic and non-genetic factors likely contributed to specify the subpopulations. The gene expression signatures might be driven by genomic profiles, including *KRAS*, and other environmental factors, including the drug treatment. The gradual reversion of drug-resistant signatures after drug withdrawal (Additional file 15) suggests that non-genetic regulatory mechanisms could be involved in the specification. To devise effective anti-cancer treatment strategies, we need to understand the underlying mechanisms whereby transcriptome heterogeneity is maintained in the tumor.

According to the prognostic value of the activating *KRAS* mutation and RS, PDX cells with *KRAS*^*G12D*^ expression and high RS would be expected to be drug resistant. Moreover, as a whole population, the PDX cells had a high *KRAS*^*G12D*^ variant allele frequency and high RS that masked the no *KRAS*^*G12D*^ (*KRAS*^*WT*^) and/or low RS cell types. The use of tumoricidal anti-cancer drugs with different mechanisms of action (cytotoxic and targeting specific signaling pathways) dramatically changed the gene expression features of the PDX cells in this study from *KRAS*^*G12D*^ plus high RS to *KRAS*^*G12D*^ plus low RS. The result was counterintuitive, since high RS is significantly associated with worse prognosis of LUAD patients. However, in an independent PDX case, cells with a low RS also survived *in vitro* anti-cancer treatments, supporting the validity of the unexpected results.

The unexpected results indicate that (1) tumor cells with activated *KRAS* signatures were drug targets, but the *KRAS* mutation itself was not a target, and (2) the actual tumor population responsible for drug resistance might be masked by dominant genomic characteristics within a bulk population. In this study, the cells that survived the effective treatments retained the *KRAS* mutation but seemed to stay in a dormant state without activating *KRAS* signaling. Interestingly, the molecular signatures of this group indicated upregulation of genes involved in the ion channel transport and P-type ATPases, which might play key roles in drug resistance [10]. Whether this potentially drug-resistant population is indeed a pre-existing tumor subclone or dynamically changes gene expression signatures in response to drug treatments needs to be addressed by future studies.

## Conclusions

This study demonstrates that gene expression and somatic SNVs of single tumor cells could be retrieved simultaneously by single-cell RNA-seq. Furthermore, the genomic data obtained could be used to elucidate potentially drug-resistant subclones and to generate hypotheses on the molecular mechanisms of treatment resistance that are masked in the whole cancer cell population.

## Materials and methods

### Patient samples and PDX cells

This study was carried out in accordance with the principles of the Declaration of Helsinki, and approved by The Samsung Medical Center (Seoul, Korea) Institutional Review Board (no. 2010-04-004). Participants in this study gave written informed consent for research and publication of the results. Surgical specimens were acquired from a 60-year-old male patient who underwent surgical resection of a 37-mm irregular primary lung lesion in the right middle lobe (LC-PT-45), and from a 57-year-old female patient who underwent surgical resection of a metachronous brain metastasis (LC-MBT-15). The LC-PT-45 tumor was taken in a treatment-naïve status whereas the LC-MBT-15 tumor was taken after standard chemotherapy and erlotinib treatments. Pathologic examination of the primary tumors revealed a poorly differentiated lung adenocarcinoma based on the World Health Organization criteria [49]. The PDX cells were isolated and cultured *in vitro* as described previously [24, 30, 50]. Briefly, surgically removed tumor tissues were directly injected into the subrenal space of 6–8-week-old humanized immunocompromised female NOG (NOD/Shi- SCID/IL-2Rγ-null) mice (Orient Bio, Seongnam, Korea). Xenograft tumors were taken from the mice for PDX cell culture and validated by short tandem repeat DNA fingerprinting as having been derived from the original tumor. We used PDX cells at fewer than three *in vitro* passages for single-cell RNA-seq and drug screening. Animal care and handling was performed according to the National Institute of Health Guide for the Care and Use of Laboratory Animals (NIH publication no.80-23, revised 1978).

### Drug screening with PDX cells

Dissociated PDX cells were cultured in neurobasal media-A supplemented with N2 (×1/2; Life Technologies, Carlsbad, CA, USA), B27 (×1/2; GIBCO, San Diego, CA, USA), basic fibroblast growth factor (bFGF; 25 ng/mL; R&D Systems, Minneapolis, MN, USA), epidermal growth factor (EGF; 25 ng/mL; R&D Systems), neuregulin 1 (NRG; 10 ng/mL; R&D Systems), and insulin-like growth factor 1(IGF1; 100 ng/mL; R&D Systems). The cells grown in these serum-free sphere culture conditions were seeded in 384-well plates (500 cells/well), and treated with a drug library (Selleck, Houston, TX, USA). The drug library was composed of targeted agents and cytotoxic chemotherapeutics, which were included in the clinical guideline or current clinical trial for the treatment of non-small cell lung cancer. After 3 days of incubation at 37 °C in a 5 % CO_2_ humidified incubator, cell viability was analyzed using an adenosine triphosphate monitoring system based on firefly luciferase (ATPlite™ 1step; PerkinElmer, Waltham, CA, USA). Test concentrations for each drug were empirically determined to produce a clinically relevant spectrum of drug activity. Dose response curves and corresponding half maximal (50 %) inhibitory concentration values (IC_50_) were calculated using the S+ Chip Analyzer (Samsung Electro-Mechanics, Suwon, Korea) [51].

### WES and data processing

Genomic DNA was extracted from PDX cells using the QIAamp® DNA Mini kit (Qiagen, Hilden, Germany) or QIAamp DNA Blood Maxi Kit (Qiagen). Exomes were captured using the SureSelect XT Human All Exon V5 kit (Agilent Technologies, Inc., Santa Clara, CA, USA). The sequencing library was constructed and analyzed by the HiSeq 2000 or 2500 systems (Illumina, San Diego, CA, USA) using the 100-bp paired-end mode of the TruSeq Rapid PE Cluster kit and TruSeq Rapid SBS kit (Illumina). Mean target coverage for exome data was 153.4 ± 26.99 × .

Exome-sequencing reads were aligned to the hg19 reference genome using BWA-0.7.10 [52]. Putative duplications were marked by Picard-1.93 software [53]. Sites potentially harboring small insertions or deletions were realigned, and SNVs were called by applying GATK-3.2 [54] ‘HaplotypeCaller’ with known variant sites identified from phase I of the 1000 Genomes Project [55] and dbSNP-137 [56], using default option parameters. Then, called variants were evaluated to obtain highly accurate call sets through a two-stage processing step of ‘VariantRecalibrator’ and ‘ApplyRecalibration’, using default option parameters. To detect somatic mutations with increased sensitivity both in lower and higher allele frequencies [57], we used the caller programs of MuTect-1.1.5 [58] and VarScan2 [59].

Estimation of copy number variation from WES was performed using the ExomeCNV software package [26] in default quantification mode. Circular binary segmentation was applied to determine the neighboring regions of DNA that exhibited a statistically significant difference in copy number. The output was also applied to infer tumor purity using AbsCNseq [27].

### Isolation of single cells and RNA-seq

We used the C1™ Single-Cell Auto Prep System (Fluidigm, San Francisco, CA, USA) with the SMARTer kit (Clontech, Mountain View, CA, USA). For the original experiment, 44 cells were captured as a single isolate on a C1 array chip for mRNA sequencing (17–25 μm) as determined by microscopic examination, and 34 passed the required criteria for cDNA quantity and quality as measured with a Qubit® 2.0 Fluorometer (Life Technologies) and 2100 Bioanalyzer (Agilent). RNA from bulk cell samples was also amplified using a SMARTer kit with 10 ng of starting material. Libraries were generated using the Nextera XT DNA Sample Prep Kit (Illumina) and sequenced on the HiSeq 2500 using the 100-bp paired-end mode of the TruSeq Rapid PE Cluster kit and TruSeq Rapid SBS kit.

### RNA-seq data processing

RNA-seq reads were aligned to the human genome reference (hg19) together with splice junction information of each sample using the two-pass default mode of STAR_2.4.0d [60]. Gene expression was quantified by implementing RSEM v.1.2.18 [61] in default mode with Genecode v.19 [62] annotation, and calculated as the sum of isoform expression. Pre-processing steps for RNA-seq reads before calling variants were optimized by deduplication, splitting reads into exon segments, hard-clipping any sequences overhanging the intronic regions, realigning reads and recalibration using GATK-3.2 [54]. Then, variants were called by ‘HaplotypeCaller’ mode with option parameters of (−R hg19.fa --genotyping_mode DISCOVERY -recoverDanglingHeads -dontUseSoftClippedBases --dbsnp dbsnp_137.hg19.vcf -stand_emit_conf 20 -stand_call_conf 20 -nct 4). Highly accurate variants were filtered by applying ‘VariantFiltration’ (option parameters: −F hg19.fa -window 35 -cluster 3 -filterName FS -filter “FS > 30.0” -filterName QD -filter “QD < 2.0” \). After removal of variant call quality Q < 20, further filtering was applied to SNVs that were considered to be potential false positives in RNA-seq by SNPiR [34]. We regarded only those SNVs which overlapped with WES as true positives. The overall process of calling and filtering the variants is summarized in Figure S4a in Additional file 7.

### Computing RS using multivariate markers

RSs were regression coefficients calculated by a linear combination of the expression values of the prognosis markers using a training set [6] of LUAD patients. Prognosis markers were also derived from the previous report [6] that classified LUAD patients according to gene expression profiles of the suggested markers, and 69 genes were ultimately chosen by overlapping our data sets after gene filtering of zero expression across all single cells. These filtered genes (Additional file 11) were validated as prognosis markers with independent LUAD datasets from The Cancer Genome Atlas [2] and from a Korean LUAD cohort [63]. Batch effects on gene expression between independent datasets were removed by means of ComBat [64]. Regression coefficients and *P* values of the training set were estimated using univariate Cox proportional hazards regression modeling and ordered by *P* values. To partition patient samples into high- and low-RS-based groups upon computation of response score, we applied a 60th percentile cutoff as described in Beer et al*.* [6]. Survival analysis was performed using the R Survival package [65] and validated through Kaplan-Meier survival curves with log-rank testing (training set, *P* = 1.04 × 10^−6^; validation set, *P* = 9.25 × 10^−3^) (Figure S8b in Additional file 12).

To classify the control and drug-treated PDX cells into semi-supervised clustered single cells (LC-PT-45, Fig. 4; LC-MBT-15, Additional file 16: Figure S12), a classification SVM type 1 (C-SVM classification) model was applied using the R package e1071 [66].

### Gene set signature activation analysis

To characterize gene expression features of a subgroup compared with the other groups among the classified single cells, we utilized the GSEA-P program with default mode searching for significantly enriched gene set signatures [67]. Applied gene sets were derived from the three major curated pathway databases of KEGG, REACTOME, and BIOCARTA in MSigDB v.4.0 [68]. To estimate the gene set activation status of a single sample, gene set variation analysis [69] was applied in default mode.

### Validating gene expression and expressed SNVs at the RNA level by qPCR

Gene expression and expressed SNVs were assessed by qPCR or SNP type PCR across single cells using a Biomark HD system (Fluidigm). cDNAs obtained from the C1 array for mRNA sequencing chip were subjected to specific target amplification following the manufacturer’s recommendations. For the gene expression qPCR, Delta Gene Assay (Fluidigm) with EvaGreen second generation dsDNA binding dye was performed for gene sets selected from the RS genes (Additional file 6). To compare correlations between RNA-seq and qPCR platforms for the selected 43 gene expression, mean fold change over median expression was calculated as in the previous study [33]. Validation of expressed SNVs at the RNA level was carried out using a SNP Type Assay (Fluidigm) with locus-specific primer sequences. Primers were designed using D3™ software (Fluidigm), and sequences are available in Additional file 6.

### Validating genomic variants at the DNA level by ddPCR

PDX cells were labeled with 6-carboxyfluorescein succinimidyl ester (Life Technologies) and sorted into single cells using a FACSAria™ III flow cytometer (BD Biosciences, CA, USA). Wells with a single green fluorescence signal were manually inspected and selected for amplification of genomic DNA with a GenomiPhi V2 DNA Amplification Kit (GE Healthcare, Little Chalfont, UK). The mutant alleles were detected using ddPCR Supermix for Probes reagents (Bio-Rad, Hercules, CA, USA) implemented using a QX200 ddPCR system, following the manufacturer’s protocols. The negative signal of droplets was normalized with a vehicle control, and the numbers of wild-type or mutation alleles in droplets were estimated in a Poisson distribution. Variant allele frequency was calculated by counting copies of mutation alleles over the total number of detected alleles. We regarded genotypes of detected variants as homozygous when the variant allele frequency was higher than 90 %. Sequences of the primers used in ddPCR are available in Additional file 6.

### Statistical analysis of single-cell gene expression

Linear regression was applied to scatter plots of the averaged single cells over the pooled-cell samples in Fig. 2a with zero intercepts. The inter-correlation distribution between single cells was calculated as Pearson’s and Spearman’s correlation coefficients, and plotted as a density plot with a kernel function fitting over the histograms (Fig. 2b). Multiple regression analysis estimated how many single cells hypothetically accounted for the pooled cell fraction. Single-cell samples were randomly chosen with the given number and the adjusted R^2^ (Fig. 2c) and the overlap ratio (Fig. 2e) were determined 1000 times with permutation. The differences in normalized RS, gene expression, and gene set activation score between single-cell subgroups were tested using two-tailed Student’s *t*-tests.

### Data access

The data reported in this paper have been deposited at the Samsung Genome Institute (SGI) data repository [70] and at the NCBI Gene Expression Omnibus (GEO) under accession number GSE69405.

## Additional files

Additional file 1: Figure S1. Propagation of LUAD tumor cells in the xenograft model. a A summarized depiction of the experimental process of tumor engraftment from a LUAD patient into mice. b Histological examination by a licensed pathologist determined the tumor area (dotted lines) in formalin-fixed, paraffin-embedded (FFPE) samples of a patient tumor. c Evaluation of propagation of LUAD from a patient and in mice by immunohistochemistry analysis, using lung adenocarcinoma cell-specific markers (TTF-1 and Napsin A) and a lung squamous cell carcinoma-specific marker (CK 5/6). Scale bar, 100 μm (b, c). *H&E* hematoxylin and eosin. Additional file 2: Table S1. Somatic mutations identified in PDX cells. Additional file 3: Table S2. Summary of mapping information for RNA-seq samples. Additional file 4: Figure S2. Coverage plots of transcripts based on expression level. Expression levels of the transcripts were rank-ordered and classified in each sample. *Top*: top 1000 transcripts. *Middle*: 500 transcripts above and 500 transcripts below the median, rank-ordered. *Bottom*: bottom 1000 transcripts. Coverage ratio was normalized to the maximal degree of coverage in each sample. Standard deviation across samples is depicted as thinner vertical lines over thicker curves. Additional file 5: Figure S3. Evaluation of batch effects using a technical replicate set. a Principal component analysis for total data sets of single cells used in this study. b, c Interrelation between single cells from LC-PT-45 and LC-PT-45-Re, a technical replicate set, in gene expression (measured by Pearson *r*) (b), and in expressed SNVs (measured by overlap ratio) (c). Unsupervised hierarchical clustering trees were constructed by applying Euclidean distance. d–f Reciprocal relations between single cells and bulk cells from the other batch set. d Scatter plots depicting average gene expression of single cells and bulk cells. *Black dotted lines* are *x = y* lines with correlation coefficients (Pearson *r* and Spearman *r*) for linear fit. e Explanatory power (adjusted R-square) of gene expression of various numbers of single cells relative to the bulk cells was determined by multiple regression analysis using randomly selected cell numbers with permutation (×1000). f Overlap ratio of expressed SNVs of various single-cell numbers relative to that of the bulk cells was calculated with a randomly selected given number of cells with permutation (×1000). For the boxplots in (e) and (f), box = interquartile range (IQR) between the first and the third quartiles, error bars = 10th–90th percentiles. g Distribution of mean expression across single cell RNA-seq data for the total genes (main graph) and for the genes used in qPCR (inset, n = 43). h Evaluation of gene expression variation across single cells between two batch sets of RNA-seq (*left*), and between the two technical platforms of RNA-seq and qPCR (*right*). For parallel comparison (left and right panels), 43 target gene probes were selected for validation. *Black dotted lines* are *x = y* lines with correlation coefficients (Pearson *r* and Spearman *r*) for linear fit. Additional file 6: Table S4. Information on primers used in qPCR (expression and genotyping) and ddPCR. Additional file 7: Figure S4. Detection and filtering of variants in single-cell RNA-seq data. a Schematic overview of data processing for the discovery of expressed variants. See “Materials and methods” for details. b Comparative evaluation of the detection processes for genomic variants in RNA-seq, following filtering steps marked in (a). Additional file 8: Figure S5. Expressed genotypes of SNVs in H358 cells. a *Top left*: bar graph of mutation events per sample. *Bottom left*: heat map of mutation profiles across samples. *Right*: bar graph of normalized mutation fraction over total single cells (n = 50). b Mapping information from RNA-seq reads to a human reference genome (hg19). Vertical bar plots of the number of RNA-seq reads (left y-axis) and scatter plots with a connecting line for the unique mapping rate (uniquely mapped reads/input reads, right y-axis) are in the same order as in (a). Additional file 9: Figure S6. Summary heatmap identifying concordance between RNA-seq and genotyping PCR across matched single cells. *Top left*: bar graph of concordance events per sample. *Bottom left*: heat map of concordance profiles across samples. Right: bar graph of normalized concordance fraction over total single cells (LC-PT-45-Re, n = 43). Additional file 10: Figure S7. Comparison of various platforms for detecting mutant single cell fractions and variant allele frequencies of bulk cells. a The summarized results of ddPCR for selected SNVs at the DNA level. *Top left*: bar graph of mutation events per sample. *Bottom left*: heat map of mutation profiles across samples. *Right*: bar graph of normalized mutation fraction over total single cells (LC-PT-45, n = 21). b Multidimensional scatter plots of the comparative fraction of SNVs across various platforms. *Black dotted lines* are *x = y* lines with correlation coefficients (Pearson *r* and Spearman *r*) for linear fit. c The variant allele frequency (*VAF*) of *KRAS* ^*G12D*^ across single cells separately measured for DNA (by ddPCR) and RNA (by RNA-seq). Additional file 11: Table S3. Prognostic genes used for computing risk scores. Additional file 12: Figure S8. Application of risk scores to patient survival in LUAD cohorts. a Strategy to classify single cells according to prognostic marker expression. b Kaplan-Meier curves of overall survival of patients in two independent LUAD cohorts and of recurrence-free survival of patients in a Korean LUAD cohort, according to the estimated risk scores (log-rank test). Additional file 13: Figure S9. Distinct gene expression signatures among the classified single cell subgroups along with the drug treatment groups. a Expression heatmap discriminating single cells into subgroups classified as in Fig. 4c. bREACTOME-defined ion channel transport is significantly activated in group 2 compared with the other groups, as determined by gene set enrichment analysis. Statistical significance was determined using the nominal *P* values. *ES* enrichment score; *NES* normalized enrichment score. Gene set activation signatures were estimated for the control and drug-treated PDX cells by gene set variation analysis. c Gene expression signature for the cell cycle was estimated by gene set variation analysis. The gene set for the cell cycle signature was obtained from REACTOME. Additional file 14: Figure S10. Procedure and the results of drug screening for LC-PT-45. a The overall process from PDX cell preparation to drug screening. b Summarized list of drugs used in the screening, their known targets, and calculated IC_50_s. The six anti-cancer compounds used in this study are indicated: ^†^cytotoxic compounds carboplatin and docetaxel; *molecular targeting compounds DAPT, BKM120, BEZ235, and selumetinib. Additional file 15: Figure S11. Assessment of phenotypic reversibility for selumetinib-mediated gene expression signatures. a The experimental design to examine the change of gene expression under selumetinib. LC-PT-45 PDX cells were serially collected before and after 3-day exposure to 1 μM selumetinib, and on 3 days (R3) and 7 days (R7) after the washout of the drug. b Normalized RSs (*top*) and adjusted-expression of the 69 prognostic markers (*middle*) with *KRAS* mutant expression (*bottom*) for the mock- and selumetinib-treated PDX cells. c–f Comparative features among the mock- and selumetinib-treated PDX cells. c *KRAS* gene expression (Log2 ratio of TPM + 1). Gene set signature scores (computed by gene set variation analysis) corresponding to the *KRAS* overexpression signature [39] (d), *KRAS* mutation signature [40] (e), and MAPK pathway signature (gene sets from BioCarta) (f). Additional file 16: Figure S12. Validation of analytical procedures on an additional PDX, LC-MBT-15. a A scatter plot of the average gene expression of single cells (n = 49) and that of the corresponding bulk cells (~1 × 10^5^ cells). *Black dotted line* is the *x = y* line with correlation coefficients (Pearson *r* and Spearman *r*) for linear fit. b Inter-correlation (Pearson *r*) between gene expression of single cells. Density plots were constructed with a kernel function fitting over the histograms. c Explanatory power (adjusted R-square) of gene expression of various numbers of single cells relative to the bulk cells was determined by multiple regression analysis using randomly selected cell numbers with permutation (×1000). d Overlap ratio of expressed SNVs among single cells. Density plots were constructed with a kernel function fitting over the histograms. e Overlap ratio of expressed SNVs of various single-cell numbers relative to that of the bulk cells was calculated with a randomly selected given number of cells with permutation (×1000). For the boxplot, box = interquartile range (IQR) between the first and the third quartiles, error bars = 10th–90th percentiles. f *Top*: bar graph of normalized RS. *Middle*: heatmap of expression of 69 prognostic markers. *Bottom*: bar graph of *KRAS* and *EGFR* mutation status of single cells. g Scatter plots demonstrating the lack of impact of the *EGFR* mutation on RSs of LC-MBT-15 single cells. Horizontal lines represent the mean. h *EGFR* gene expression (Log2 ratio of TPM + 1). For the boxplots in (g, h), box = IQR between the first and the third quartiles, error bars = 10th–90th percentiles. i Graphical illustration of principal component analysis of the genes discriminating between the low-RS and high-RS subgroups. Ellipsoids were generated with standard deviations around each subgroup. j *Top*: bar graph of normalized RSs. *Middle*: heatmap of adjusted-expression of the 69 prognostic markers. *Bottom*: *KRAS* and *EGFR* mutation status for the control and drug-treated PDX cells. k Gene set activation signatures were estimated for single cells (*left*) and the control and drug-treated PDX cells (*right*) by gene set variation analysis. Gene expression signatures for ion channel transport and cell cycle were from REACTOME. l Results from the principal component (PC) analysis on single cells along with the control and drug-treated PDX cells. Ellipsoids corresponding to the single cell subgroups [low-RS (*green*), high-RS (*red*)], with the control and drug-treated PDX cells projected on the *PC1*-*PC2* plane. Using single cell subgroups as a training set, a C-SVM classification was applied to a test set of the control and drug-treated PDX cells. Additional file 17: Figure S13 The results of drug screening for LC-MBT-15. Summarized list of drugs used in the screening, their known targets, and calculated IC_50_s. The six anti-cancer compounds used in this study are indicated: ^†^cytotoxic compounds carboplatin and docetaxel; *molecular targeting compounds afatinib, DAPT, erlotinib, and tivantinib).

## Abbreviations

bp: base pair
ddPCR: droplet digital PCR
LUAD: lung adenocarcinoma
MAPK: mitogen-activated protein kinase
PCA: principal component analysis
PCR: polymerase chain reaction
PDX: patient-derived xenograft
PI3K: phosphatidylinositide 3-kinase
qPCR: quantitative PCR
RNA-seq: RNA sequencing
RS: risk score
RTK: receptor tyrosine kinase
SNV: single-nucleotide variation
SVM: support vector machine
TPM: transcripts per million
WES: whole-exome sequencing
WT: wild type
