# Supplementary figures and images for: Single-cell mRNA sequencing identifies subclonal heterogeneity in anti-cancer drug responses of lung adenocarcinoma cells
Source: Genome Biol. 2015 Jun 19;16(1):127. doi: 10.1186/s13059-015-0692-3 (PMC4506401; doi:10.1186/s13059-015-0692-3)

A

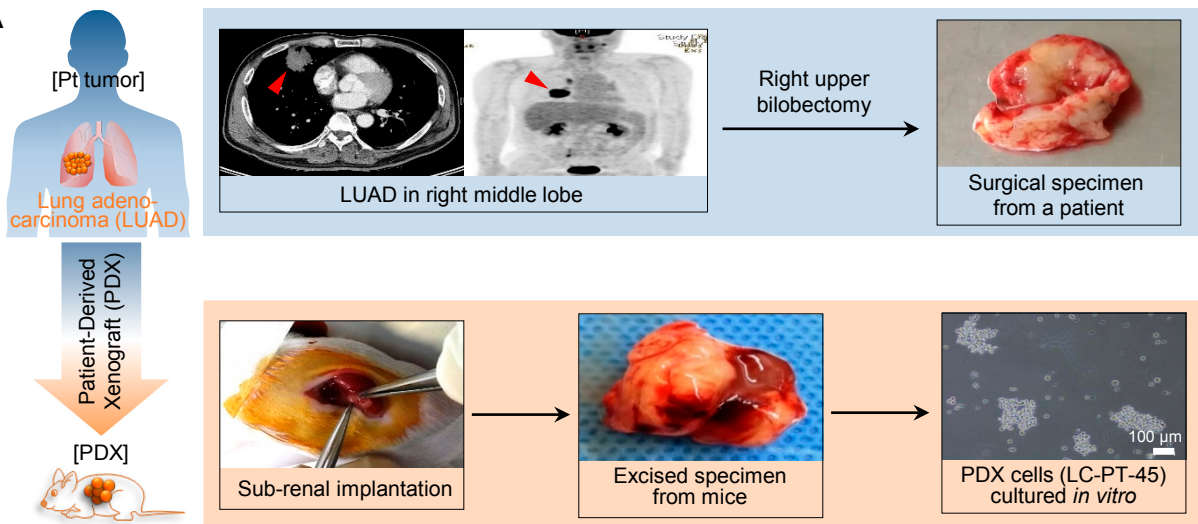

B

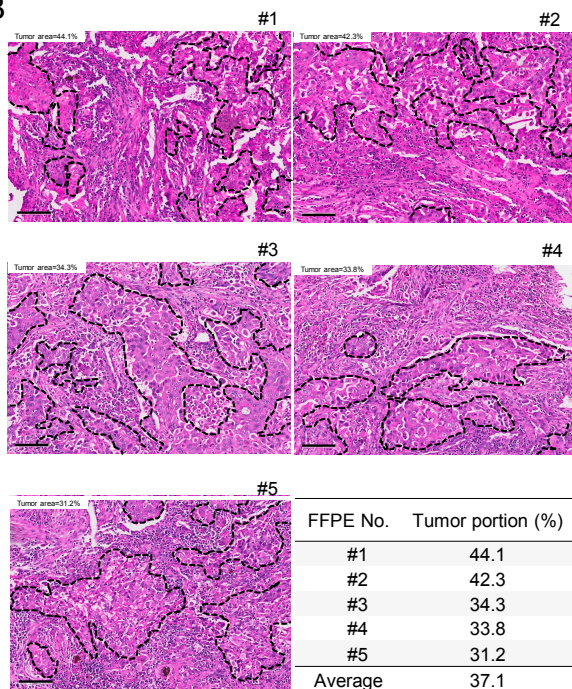

C

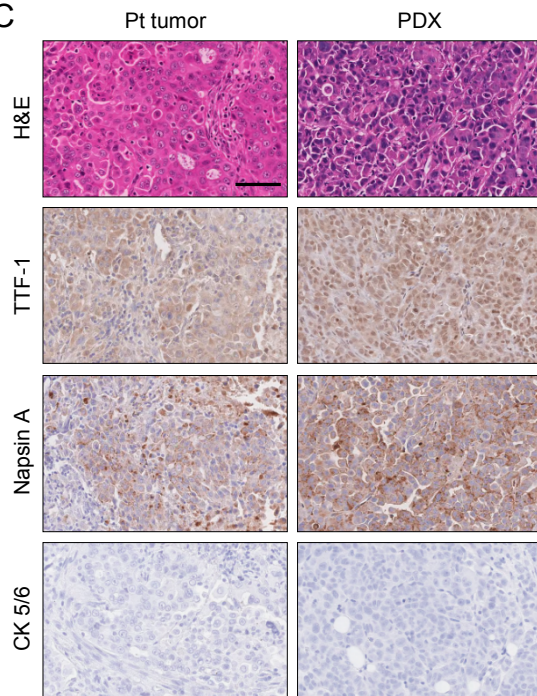

Supplement: Additional file 1: Figure S1. — Propagation of LUAD tumor cells in the xenograft model. a A summarized depiction of the experimental process of tumor engraftment from a LUAD patient into mice. b Histological examination by a licensed pathologist determined the tumor area (dotted lines) in formalin-fixed, paraffin-embedded (FFPE) samples of a patient tumor. c Evaluation of propagation of LUAD from a patient and in mice by immunohistochemistry analysis, using lung adenocarcinoma cell-specific markers (TTF-1 and Napsin A) and a lung squamous cell carcinoma-specific marker (CK 5/6). Scale bar, 100 μm (b, c). H&E hematoxylin and eosin. [file 13059_2015_692_MOESM1_ESM.pdf]

H358

LC-PT-45

LC-PT-45-Re

LC-MBT-15

Top 1000  
transcripts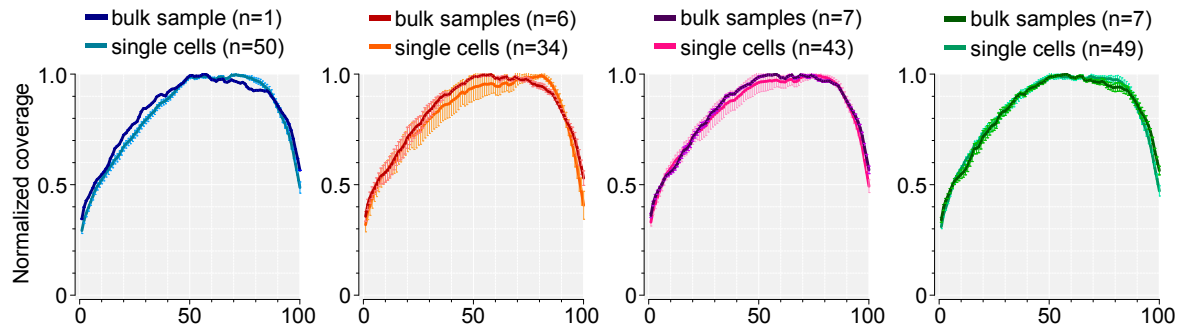Middle 1000  
transcripts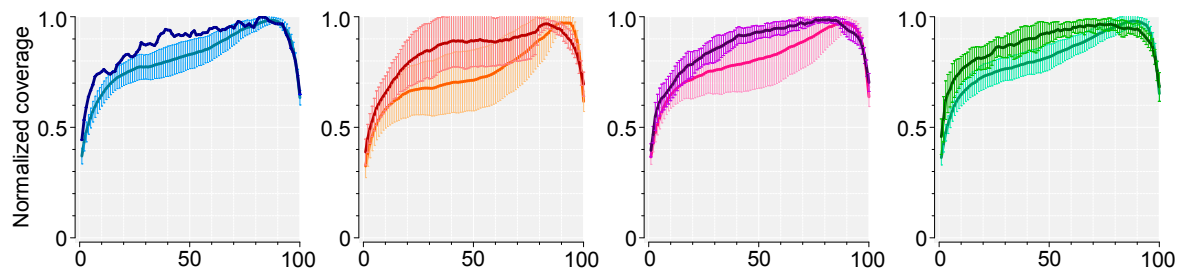Bottom 1000  
transcripts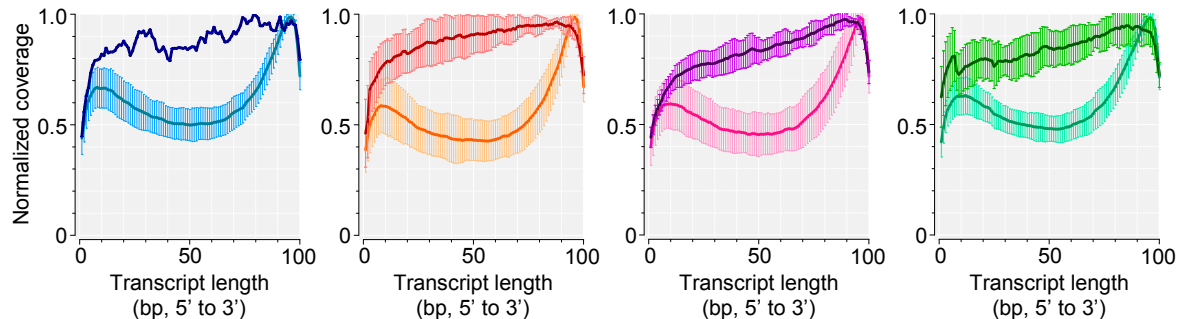

Supplement: Additional file 4: Figure S2. — Coverage plots of transcripts based on expression level. Expression levels of the transcripts were rank-ordered and classified in each sample. Top: top 1000 transcripts. Middle: 500 transcripts above and 500 transcripts below the median, rank-ordered. Bottom: bottom 1000 transcripts. Coverage ratio was normalized to the maximal degree of coverage in each sample. Standard deviation across samples is depicted as thinner vertical lines over thicker curves. [file 13059_2015_692_MOESM4_ESM.pdf]

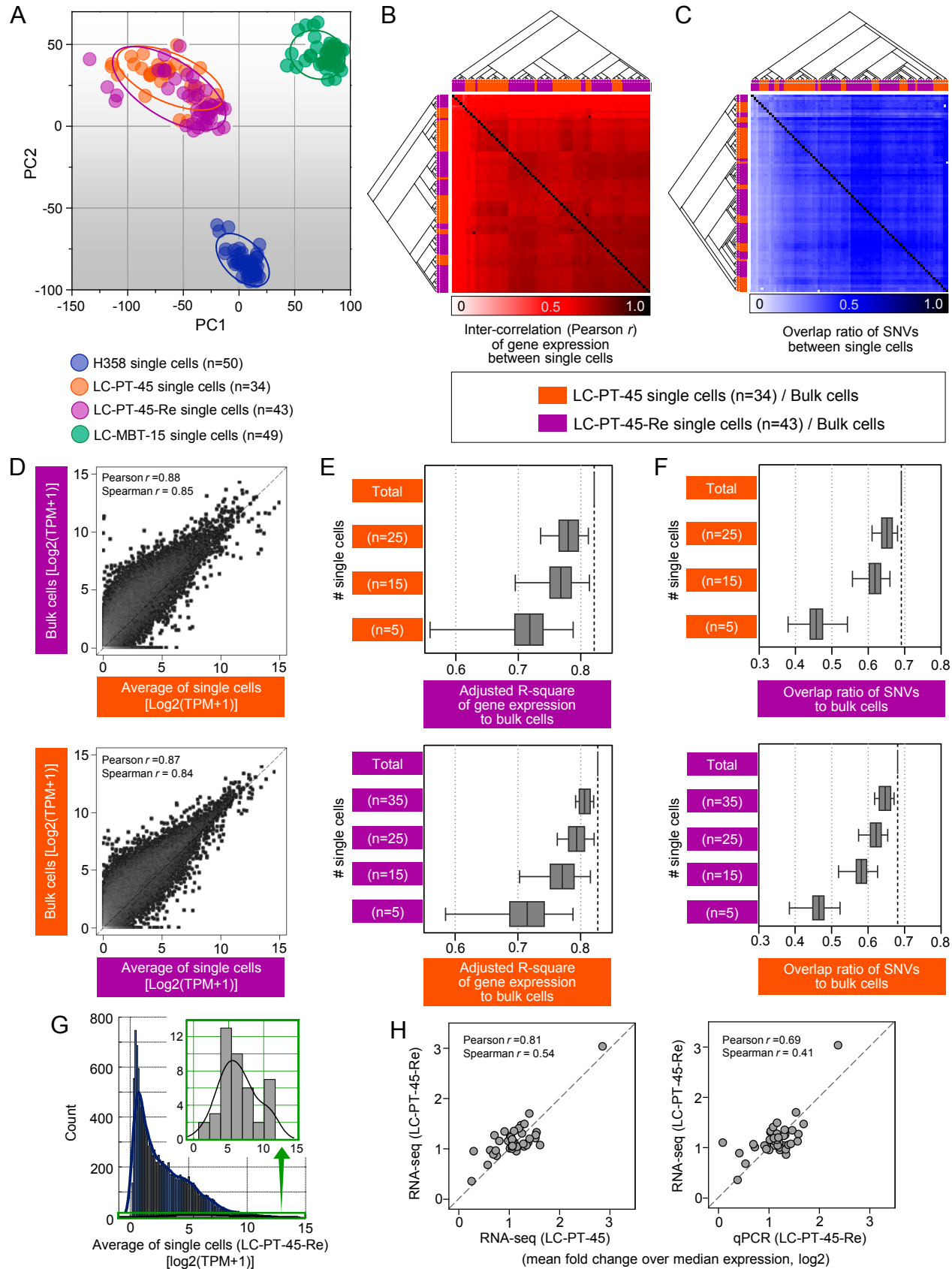

Supplement: Additional file 5: Figure S3. — Evaluation of batch effects using a technical replicate set. a Principal component analysis for total data sets of single cells used in this study. b, c Interrelation between single cells from LC-PT-45 and LC-PT-45-Re, a technical replicate set, in gene expression (measured by Pearson r) (b), and in expressed SNVs (measured by overlap ratio) (c). Unsupervised hierarchical clustering trees were constructed by applying Euclidean distance. d–f Reciprocal relations between single cells and bulk cells from the other batch set. d Scatter plots depicting average gene expression of single cells and bulk cells. Black dotted lines are x = y lines with correlation coefficients (Pearson r and Spearman r) for linear fit. e Explanatory power (adjusted R-square) of gene expression of various numbers of single cells relative to the bulk cells was determined by multiple regression analysis using randomly selected cell numbers with permutation (×1000). f Overlap ratio of expressed SNVs of various single-cell numbers relative to that of the bulk cells was calculated with a randomly selected given number of cells with permutation (×1000). For the boxplots in (e) and (f), box = interquartile range (IQR) between the first and the third quartiles, error bars = 10th–90th percentiles. g Distribution of mean expression across single cell RNA-seq data for the total genes (main graph) and for the genes used in qPCR (inset, n = 43). h Evaluation of gene expression variation across single cells between two batch sets of RNA-seq (left), and between the two technical platforms of RNA-seq and qPCR (right). For parallel comparison (left and right panels), 43 target gene probes were selected for validation. Black dotted lines are x = y lines with correlation coefficients (Pearson r and Spearman r) for linear fit. [file 13059_2015_692_MOESM5_ESM.pdf]

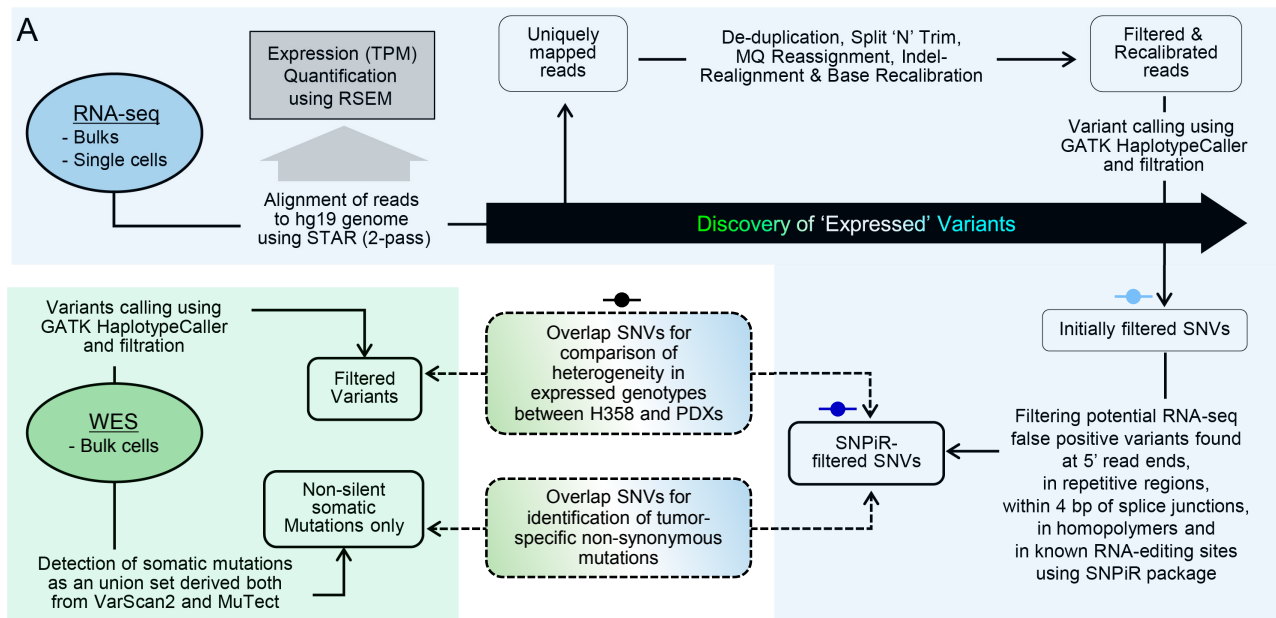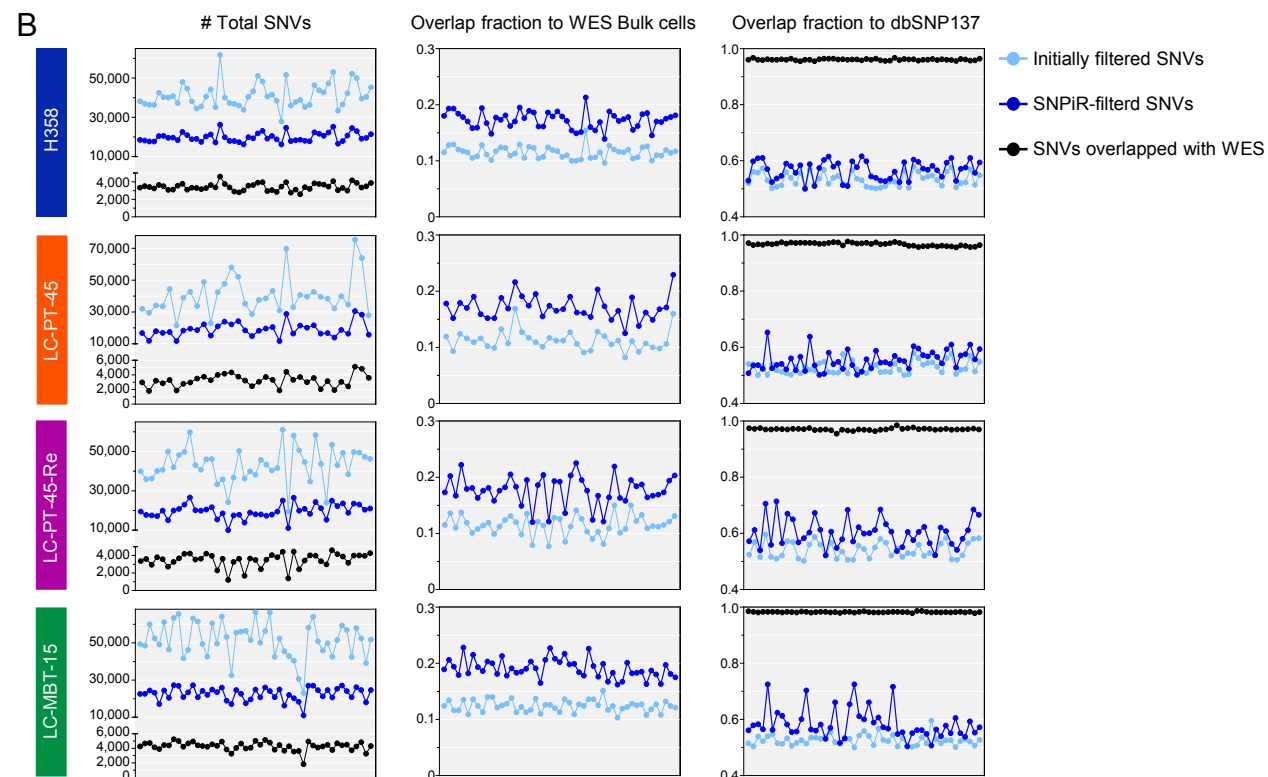

Supplement: Additional file 7: Figure S4. — Detection and filtering of variants in single-cell RNA-seq data. a Schematic overview of data processing for the discovery of expressed variants. See “Materials and methods” for details. b Comparative evaluation of the detection processes for genomic variants in RNA-seq, following filtering steps marked in (a). [file 13059_2015_692_MOESM7_ESM.pdf]

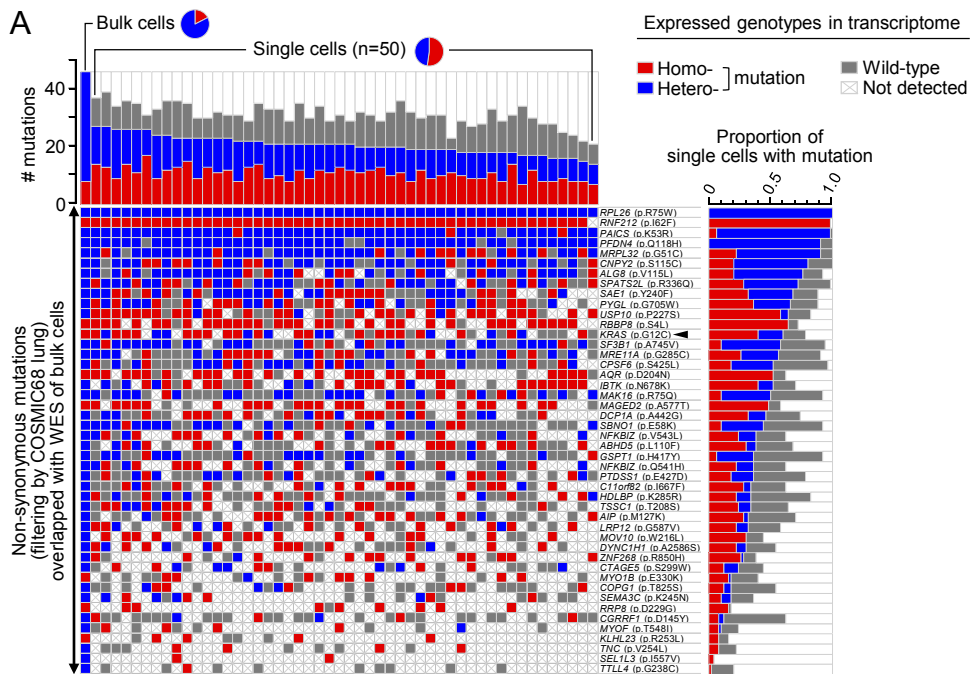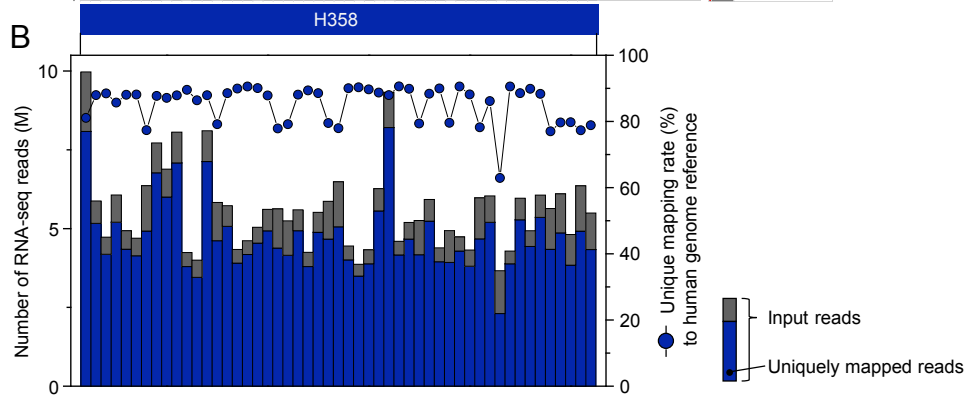

Supplement: Additional file 8: Figure S5. — Expressed genotypes of SNVs in H358 cells. a Top left: bar graph of mutation events per sample. Bottom left: heat map of mutation profiles across samples. Right: bar graph of normalized mutation fraction over total single cells (n = 50). b Mapping information from RNA-seq reads to a human reference genome (hg19). Vertical bar plots of the number of RNA-seq reads (left y-axis) and scatter plots with a connecting line for the unique mapping rate (uniquely mapped reads/input reads, right y-axis) are in the same order as in (a). [file 13059_2015_692_MOESM8_ESM.pdf]

A

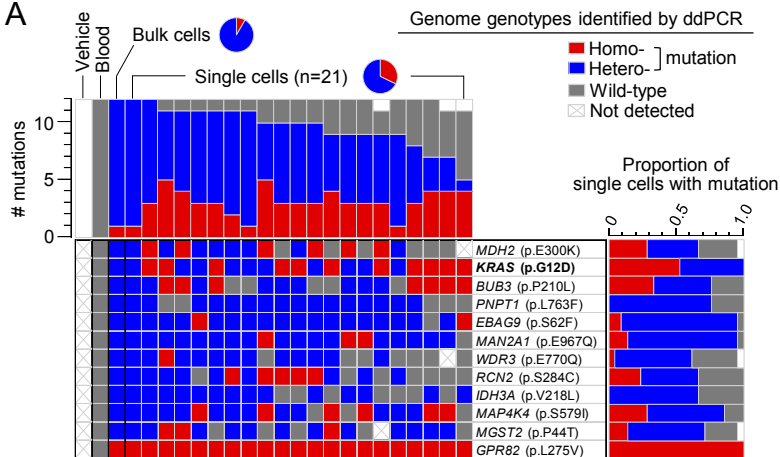

C

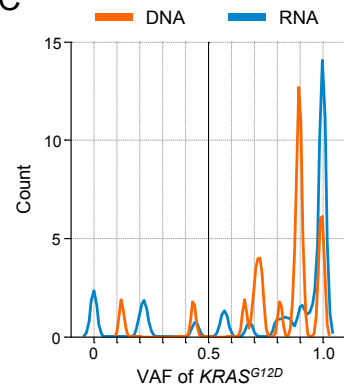

B

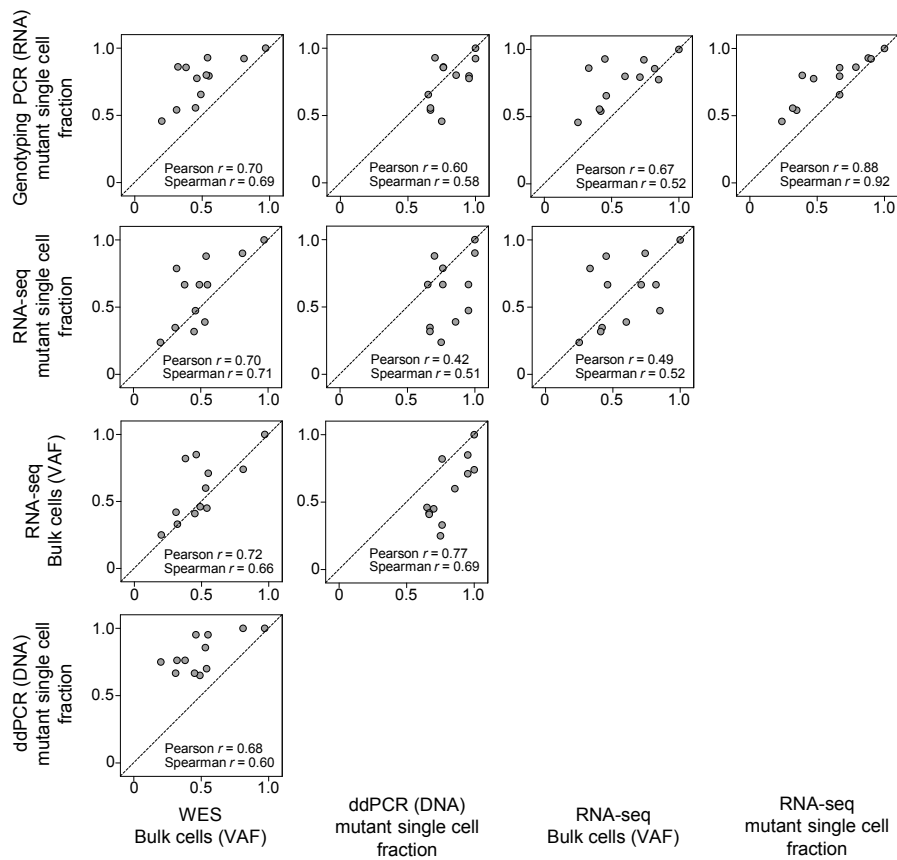

Supplement: Additional file 10: Figure S7. — Comparison of various platforms for detecting mutant single cell fractions and variant allele frequencies of bulk cells. a The summarized results of ddPCR for selected SNVs at the DNA level. Top left: bar graph of mutation events per sample. Bottom left: heat map of mutation profiles across samples. Right: bar graph of normalized mutation fraction over total single cells (LC-PT-45, n = 21). b Multidimensional scatter plots of the comparative fraction of SNVs across various platforms. Black dotted lines are x = y lines with correlation coefficients (Pearson r and Spearman r) for linear fit. c The variant allele frequency (VAF) of KRAS G12D across single cells separately measured for DNA (by ddPCR) and RNA (by RNA-seq). [file 13059_2015_692_MOESM10_ESM.pdf]

A

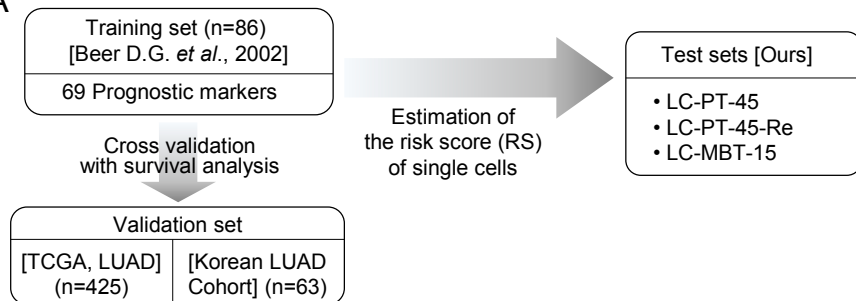

B

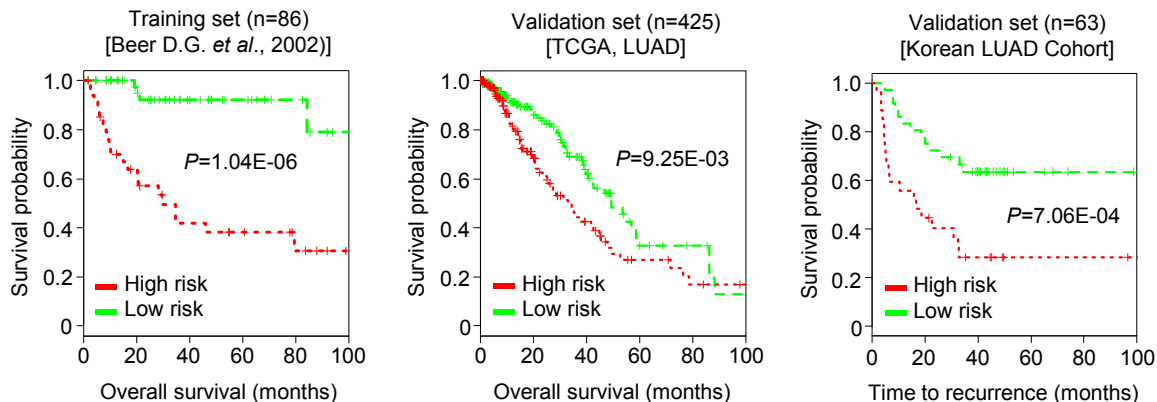

Supplement: Additional file 12: Figure S8. — Application of risk scores to patient survival in LUAD cohorts. a Strategy to classify single cells according to prognostic marker expression. b Kaplan-Meier curves of overall survival of patients in two independent LUAD cohorts and of recurrence-free survival of patients in a Korean LUAD cohort, according to the estimated risk scores (log-rank test). [file 13059_2015_692_MOESM12_ESM.pdf]

**A**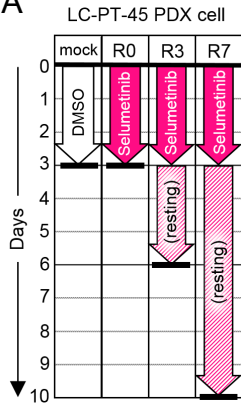**B**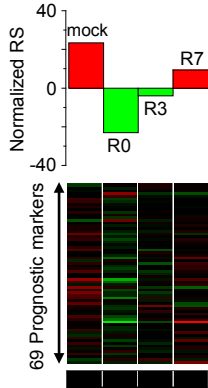**C**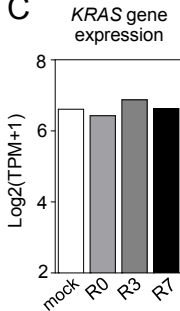**D**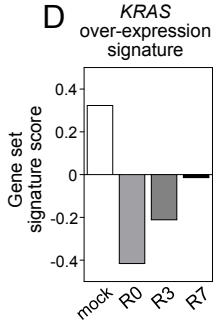**E**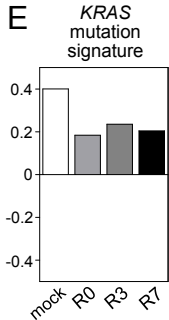**F**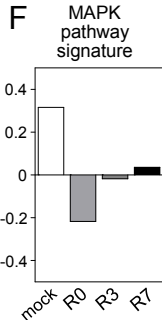

Supplement: Additional file 15: Figure S11. — Assessment of phenotypic reversibility for selumetinib-mediated gene expression signatures. a The experimental design to examine the change of gene expression under selumetinib. LC-PT-45 PDX cells were serially collected before and after 3-day exposure to 1 μM selumetinib, and on 3 days (R3) and 7 days (R7) after the washout of the drug. b Normalized RSs (top) and adjusted-expression of the 69 prognostic markers (middle) with KRAS mutant expression (bottom) for the mock- and selumetinib-treated PDX cells. c–f Comparative features among the mock- and selumetinib-treated PDX cells. c KRAS gene expression (Log2 ratio of TPM + 1). Gene set signature scores (computed by gene set variation analysis) corresponding to the KRAS overexpression signature [39] (d), KRAS mutation signature [40] (e), and MAPK pathway signature (gene sets from BioCarta) (f). [file 13059_2015_692_MOESM15_ESM.pdf]

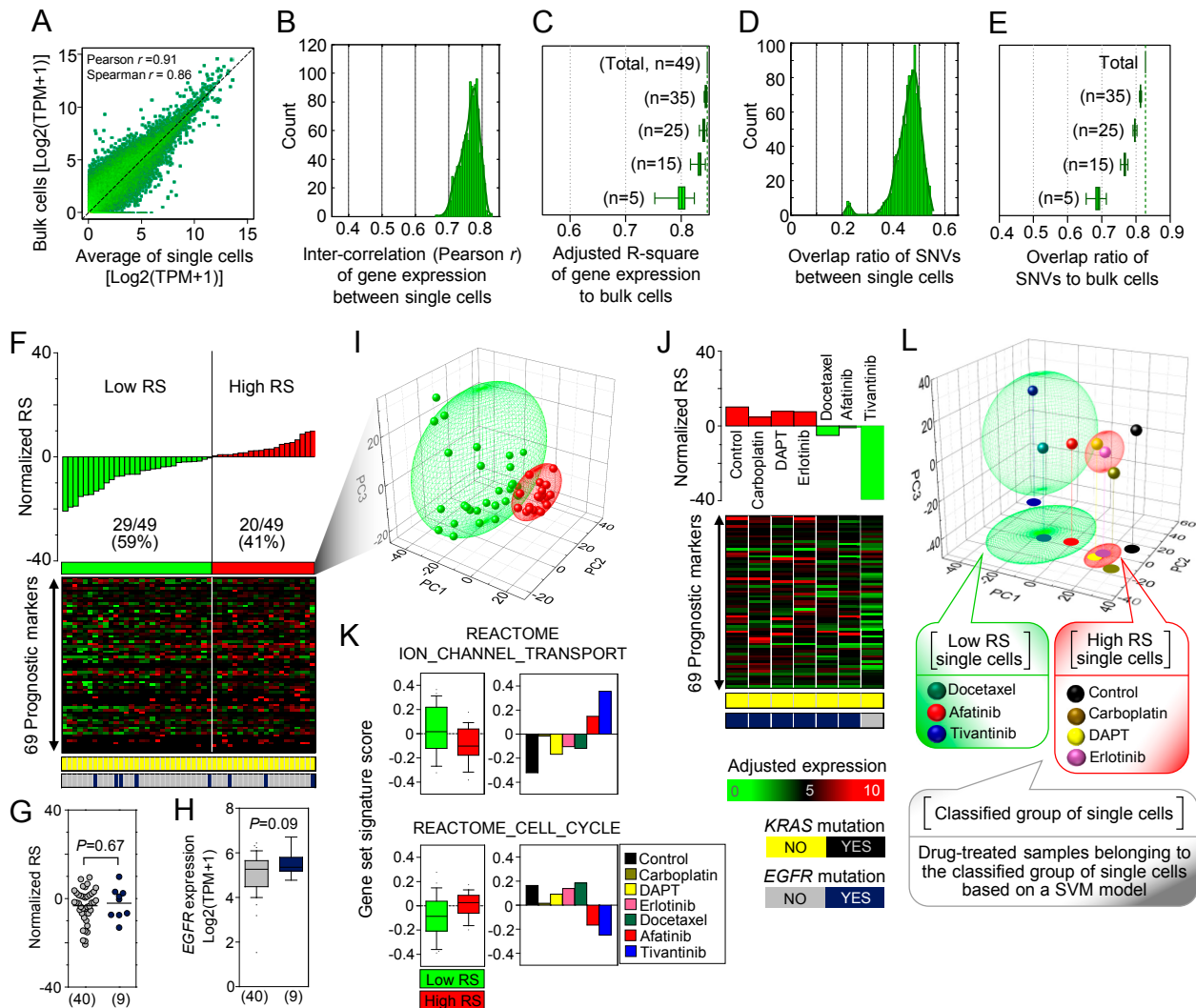

Supplement: Additional file 16: Figure S12. — Validation of analytical procedures on an additional PDX, LC-MBT-15. a A scatter plot of the average gene expression of single cells (n = 49) and that of the corresponding bulk cells (~1 × 10^5^ cells). Black dotted line is the x = y line with correlation coefficients (Pearson r and Spearman r) for linear fit. b Inter-correlation (Pearson r) between gene expression of single cells. Density plots were constructed with a kernel function fitting over the histograms. c Explanatory power (adjusted R-square) of gene expression of various numbers of single cells relative to the bulk cells was determined by multiple regression analysis using randomly selected cell numbers with permutation (×1000). d Overlap ratio of expressed SNVs among single cells. Density plots were constructed with a kernel function fitting over the histograms. e Overlap ratio of expressed SNVs of various single-cell numbers relative to that of the bulk cells was calculated with a randomly selected given number of cells with permutation (×1000). For the boxplot, box = interquartile range (IQR) between the first and the third quartiles, error bars = 10th–90th percentiles. f Top: bar graph of normalized RS. Middle: heatmap of expression of 69 prognostic markers. Bottom: bar graph of KRAS and EGFR mutation status of single cells. g Scatter plots demonstrating the lack of impact of the EGFR mutation on RSs of LC-MBT-15 single cells. Horizontal lines represent the mean. h EGFR gene expression (Log2 ratio of TPM + 1). For the boxplots in (g, h), box = IQR between the first and the third quartiles, error bars = 10th–90th percentiles. i Graphical illustration of principal component analysis of the genes discriminating between the low-RS and high-RS subgroups. Ellipsoids were generated with standard deviations around each subgroup. j Top: bar graph of normalized RSs. Middle: heatmap of adjusted-expression of the 69 prognostic markers. Bottom: KRAS and EGFR mutation status for the control and drug-treated PDX cells [file 13059_2015_692_MOESM16_ESM.pdf]
