# Supplementary material for: Single-cell mRNA sequencing identifies subclonal heterogeneity in anti-cancer drug responses of lung adenocarcinoma cells
Source: Genome Biol. 2015 Jun 19;16(1):127. doi: 10.1186/s13059-015-0692-3 (PMC4506401; doi:10.1186/s13059-015-0692-3)
Supplement: Additional file 9: Figure S6. — Summary heatmap identifying concordance between RNA-seq and genotyping PCR across matched single cells. Top left: bar graph of concordance events per sample. Bottom left: heat map of concordance profiles across samples. Right: bar graph of normalized concordance fraction over total single cells (LC-PT-45-Re, n = 43). [file 13059_2015_692_MOESM9_ESM.pdf]

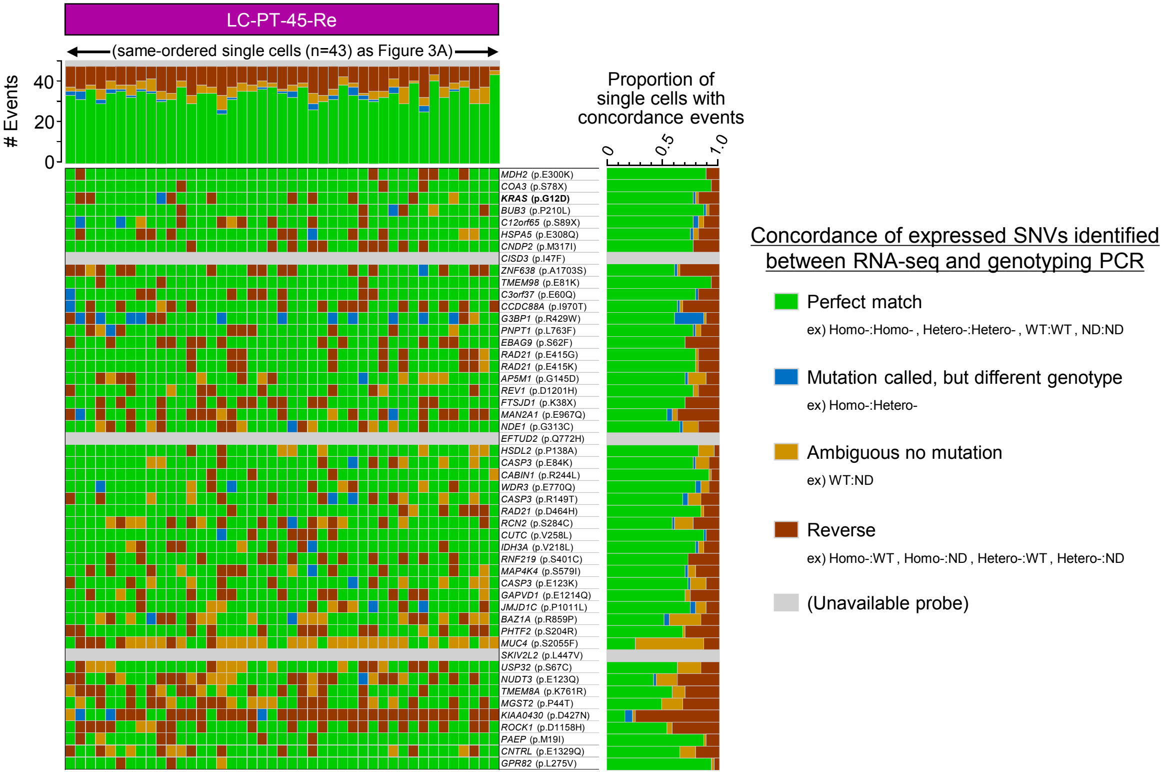
LC-PT-45-Re
(same-ordered single cells (n=43) as Figure 3A)
Events
40
20
0
Proportion of single cells with concordance events
0
0.5
1.0
MDH2 (p.E300K)
COA3 (p.S78X)
KRAS (p.G12D)
BUB3 (p.P210L)
C12orf65 (p.S89X)
HSPA5 (p.E308Q)
CNDP2 (p.M317I)
CISD3 (p.I47F)
ZNF638 (p.A1703S)
TMEM98 (p.E81K)
C3orf37 (p.E60Q)
CCDC88A (p.I970T)
G3BP1 (p.R429W)
PNPT1 (p.L763F)
EBAG9 (p.S62F)
RAD21 (p.E415G)
RAD21 (p.E415K)
AP5M1 (p.G145D)
REV1 (p.D1201H)
FTSJD1 (p.K38X)
MAN2A1 (p.E967Q)
NDE1 (p.G313C)
EFTUD2 (p.Q772H)
HSDL2 (p.P138A)
CASP3 (p.E84K)
CABIN1 (p.R244L)
WDR3 (p.E770Q)
CASP3 (p.R149T)
RAD21 (p.D464H)
RCN2 (p.S284C)
CUTC (p.V258L)
IDH3A (p.V218L)
RNF219 (p.S401C)
MAP4K4 (p.S579I)
CASP3 (p.E123K)
GAPVD1 (p.E1214Q)
JMJD1C (p.P1011L)
BAZ1A (p.R859P)
PHTF2 (p.S204R)
MUC4 (p.S2055F)
SKIV2L2 (p.L447V)
USP32 (p.S67C)
NUDT3 (p.E123Q)
TMEM8A (p.K761R)
MGST2 (p.P44T)
KIAA0430 (p.D427N)
ROCK1 (p.D1158H)
PAEP (p.M19I)
CNTRL (p.E1329Q)
GPR82 (p.L275V)
Concordance of expressed SNVs identified between RNA-seq and genotyping PCR
Perfect match
ex) Homo-:Homo- , Hetero-:Hetero- , WT:WT , ND:ND
Mutation called, but different genotype
ex) Homo-:Hetero-
Ambiguous no mutation
ex) WT:ND
Reverse
ex) Homo-:WT , Homo-:ND , Hetero-:WT , Hetero-:ND
(Unavailable probe)
