# Supplementary material for: Single-cell mRNA sequencing identifies subclonal heterogeneity in anti-cancer drug responses of lung adenocarcinoma cells
Source: Genome Biol. 2015 Jun 19;16(1):127. doi: 10.1186/s13059-015-0692-3 (PMC4506401; doi:10.1186/s13059-015-0692-3)
Supplement: Additional file 13: Figure S9. — Distinct gene expression signatures among the classified single cell subgroups along with the drug treatment groups. a Expression heatmap discriminating single cells into subgroups classified as in Fig. 4c. bREACTOME-defined ion channel transport is significantly activated in group 2 compared with the other groups, as determined by gene set enrichment analysis. Statistical significance was determined using the nominal P values. ES enrichment score; NES normalized enrichment score. Gene set activation signatures were estimated for the control and drug-treated PDX cells by gene set variation analysis. c Gene expression signature for the cell cycle was estimated by gene set variation analysis. The gene set for the cell cycle signature was obtained from REACTOME. [file 13059_2015_692_MOESM13_ESM.pdf]

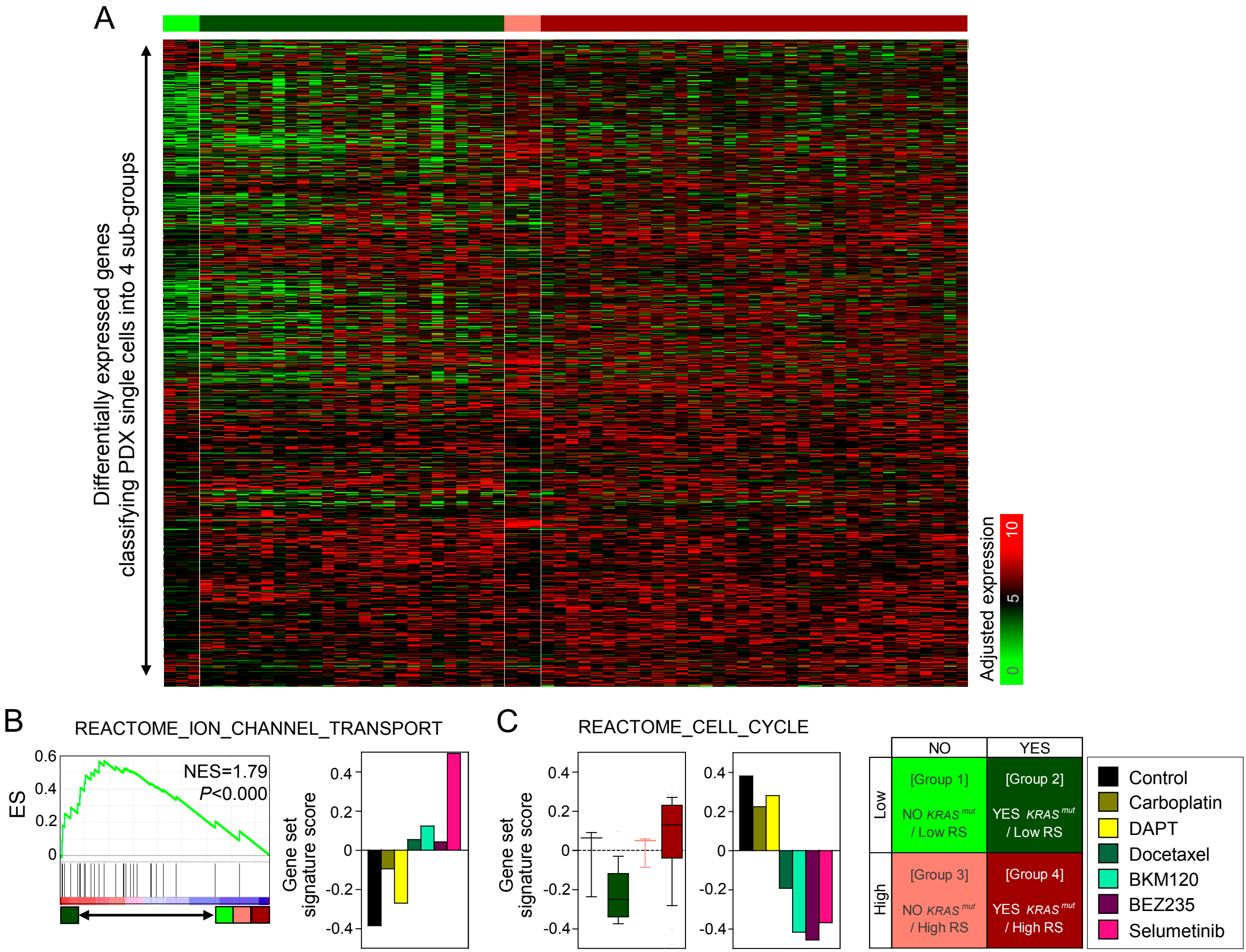
A
Differentially expressed genes classifying PDX single cells into 4 sub-groups
Adjusted expression
10
5
0
B
REACTOME_ION_CHANNEL_TRANSPORT
ES
NES=1.79
P<0.000
Gene set signature score
C
REACTOME_CELL_CYCLE
Gene set signature score
NO
YES
Low
High
[Group 1]
NO KRAS mut / Low RS
[Group 2]
YES KRAS mut / Low RS
[Group 3]
NO KRAS mut / High RS
[Group 4]
YES KRAS mut / High RS
Control
Carboplatin
DAPT
Docetaxel
BKM120
BEZ235
Selumetinib
