# Supplementary material for: Single-cell mRNA sequencing identifies subclonal heterogeneity in anti-cancer drug responses of lung adenocarcinoma cells
Source: Genome Biol. 2015 Jun 19;16(1):127. doi: 10.1186/s13059-015-0692-3 (PMC4506401; doi:10.1186/s13059-015-0692-3)
Supplement: Additional file 14: Figure S10. — Procedure and the results of drug screening for LC-PT-45. a The overall process from PDX cell preparation to drug screening. b Summarized list of drugs used in the screening, their known targets, and calculated IC50s. The six anti-cancer compounds used in this study are indicated: †cytotoxic compounds carboplatin and docetaxel; *molecular targeting compounds DAPT, BKM120, BEZ235, and selumetinib. [file 13059_2015_692_MOESM14_ESM.pdf]

A

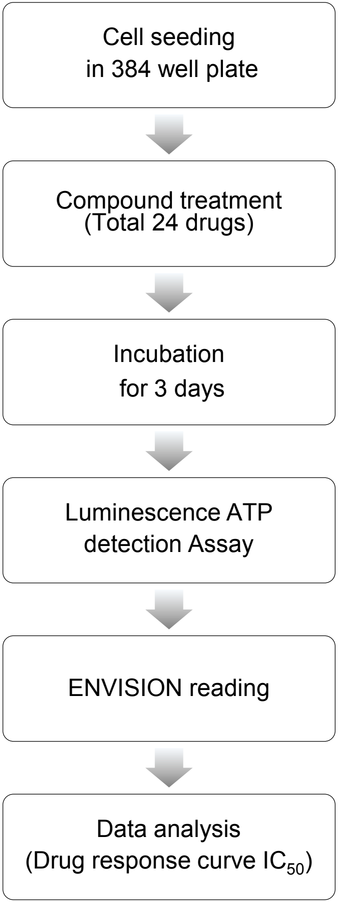

B

Drug	Target	IC$_{50}$ (nM)
BEZ235*	*PI3K*, *mTOR*	69
BKM120*	*PI3K*	240
Cabozatinib	*VEGFR2*	2,900
Carboplatin[†]	DNA synthesis	4,800
Crizotinib	*C-Met/ALK*	730
DAPT*	γ-secretase	> 20,000
Docetaxel[†]	microtubule	0.2
Dovitinib	*FLT3/c-Kit, FGFR1/3, VEGFR1-4*	750
Erlotinib	*EGFR*	3,100
Etoposide	topoisomerase II	440
Gefitinib	*EGFR*	1,600
Gemcitabine	DNA synthesis	0.5
Irinotecan	topoisomerase I	230
Lapatinib	*EGFR/HER2*	2,700
Nintedanib	*VEGFR1/2/3, FGFR1/2/3, PDGFRα/β*	> 20,000
Paclitaxel	microtubule	1.5
Pazopanib	*VEGFR1/2/3, PDGFR, FGFR, c-Kit, c-Fms*	6,500
Pemetrexed	folate and metabolite	340
Saracatinib	*SRC, ABL*	2,700
Selumetinib*	*MEK*	640
Sorafenib	multi-targeted RTK (*RAF-1/B-RAF/VEGFR2*)	2,100
Sunitinib	multi-targeted RTK (*VEGFR2/PDGFRβ*)	2,400
Temsirolimus	*mTOR, HIF-1/2, VEGF*	7,800
Vemufafenib	*B-RAF* (p.V600E)	3,000
Vinblastine	microtubule	0.5
