# Supplementary material for: Single-cell mRNA sequencing identifies subclonal heterogeneity in anti-cancer drug responses of lung adenocarcinoma cells
Source: Genome Biol. 2015 Jun 19;16(1):127. doi: 10.1186/s13059-015-0692-3 (PMC4506401; doi:10.1186/s13059-015-0692-3)
Supplement: Additional file 17: Figure S13 — The results of drug screening for LC-MBT-15. Summarized list of drugs used in the screening, their known targets, and calculated IC50s. The six anti-cancer compounds used in this study are indicated: †cytotoxic compounds carboplatin and docetaxel; *molecular targeting compounds afatinib, DAPT, erlotinib, and tivantinib). [file 13059_2015_692_MOESM17_ESM.pdf]

Drug	Target	IC_{50} (nM)
Afatinib*	*EGFR*/*HER2*	270
BKM120	*PI3K*	1000
Cabozantinib	*VEGFR2*	> 20000
Carboplatin†	DNA synthesis	> 10000
Crizotinib	*C-Met/ALK*	1900
DAPT*	γ-secretase	> 20000
Docetaxel†	microtubule	0.098
Dovitinib	*FLT3*/*c-Kit, FGFR1*/*3, VEGFR1-4*	1700
Erlotinib*	*EGFR*	> 20000
Etoposide	topoisomerase II	> 10000
Everolimus	*mTOR*	4100
Foretinib	*C-Met/VEGFR-2*	1200
Gefitinib	*EGFR*	7900
Gemcitabine	DNA synthesis	> 10000
Irinotecan	topoisomerase I	16000
Lapatinib	*EGFR*/*HER2*	3400
Nintedanib	*VEGFR1*/*2*/*3, FGFR1*/*2*/*3, PDGFRα*/*β*	2100
Paclitaxel	microtubule	2.1
Pazopanib	*VEGFR1*/*2*/*3, PDGFR, FGFR, c-Kit, c-Fms*	14000
Pemetrexed	folate and metabolite	610
Selumetinib	*MEK*	1500
Sorafenib	multi-targeted RTK (*RAF-1*/*B-RAF*/*VEGFR2*)	4000
Sunitinib	multi-targeted RTK (*VEGFR2*/*PDGFRβ*)	3900
Temsirolimus	*mTOR, HIF-1*/*2, VEGF*	570
Tivantinib*	*MET*	370
Vandetanib	*EGFR*/*VEGF*/*RET*	5700
Vemurafenib	*B-RAF* (p.V600E)	9200
Vinblastine	microtubule	0.8
